# Bacterial Temporal Dynamics Enable Optimal Design of Antibiotic Treatment

**DOI:** 10.1371/journal.pcbi.1004201

**Published:** 2015-04-23

**Authors:** Hannah R. Meredith, Allison J. Lopatkin, Deverick J. Anderson, Lingchong You

**Affiliations:** 1 Department of Biomedical Engineering, Duke University, Durham, North Carolina, United States of America; 2 Division of Infectious Diseases, Department of Medicine, Duke University Medical Center, Durham, North Carolina, United States of America; 3 Duke Infection Control Outreach Network, Duke University Medical Center, Durham, North Carolina, United States of America; 4 Center for Systems Biology, Duke University, Durham, North Carolina, United States of America; 5 Center for Genomic and Computational Biology, Duke University, Durham, North Carolina, United States of America; Rice University, UNITED STATES

## Abstract

There is a critical need to better use existing antibiotics due to the urgent threat of antibiotic resistant bacteria coupled with the reduced effort in developing new antibiotics. β-lactam antibiotics represent one of the most commonly used classes of antibiotics to treat a broad spectrum of Gram-positive and -negative bacterial pathogens. However, the rise of extended spectrum β-lactamase (ESBL) producing bacteria has limited the use of β-lactams. Due to the concern of complex drug responses, many β-lactams are typically ruled out if ESBL-producing pathogens are detected, even if these pathogens test as susceptible to some β-lactams. Using quantitative modeling, we show that β-lactams could still effectively treat pathogens producing low or moderate levels of ESBLs when administered properly. We further develop a metric to guide the design of a dosing protocol to optimize treatment efficiency for any antibiotic-pathogen combination. Ultimately, optimized dosing protocols could allow reintroduction of a repertoire of first-line antibiotics with improved treatment outcomes and preserve last-resort antibiotics.

## Author Summary

Antibiotic resistance is a growing problem that the World Health Organization describes as “one of the top three threats to global health.” To date, bacteria have developed resistance to all antibiotics used in clinical settings. Unfortunately, the evolution of antibiotic resistant bacteria is accelerating, as antibiotics continue to be misused and overused. As the antibiotic pipeline is drying up, it becomes increasingly critical to utilize the antibiotics already on the market more effectively. The key to designing better regimens lies in the ability to predict how bacteria will respond to a particular antibiotic treatment. Because of this, we need a simple metric that characterizes this pathogen-antibiotic interaction that can be easily measured and used to design dosing protocols that will effectively clear an infection. To help guide the design of effective protocols, we use quantitative modeling to develop a metric that is easy to measure and quantifies the pathogen-antibiotic interaction. Through optimized antibiotic regimens, our strategy could extend the use of first-line antibiotics, improve treatment outcome, and preserve last-resort antibiotics.

## Introduction

Bacteria eventually develop resistance to all antibiotics they encounter [1–3]. Unfortunately, the evolution of antibiotic resistant bacteria is accelerating due to the widespread use of antibiotics [4,5]. As the antibiotic pipeline is drying up and the threat of antibiotic resistance is becoming more urgent [6,7], it is critical that we better utilize the antibiotics already on the market [8–10].

One of the largest and most commonly used classes of antibiotics for treating both Gram-positive and Gram-negative bacteria is the β-lactams [11–13]. Many β-lactams, such as penicillin V, amoxicillin, and first-generation cephalosporins, are first-line antibiotics; they are recommended for initial therapy because they are highly effective against non-resistant pathogens, have a lower risk of side effects, and are less expensive, relative to second-line antibiotics [14–16]. However, the rapid emergence of extended spectrum β-lactamase (ESBL) producing pathogens has greatly limited the use of β-lactam antibiotics [13,17]. ESBL-producing pathogens have significant adverse effects on clinical outcomes due to their ability to hydrolyze penicillins, broad-spectrum cephalosporins, and monobactams [6,18,19]. Patients infected with ESBL-producing pathogens have worse prognoses and, if given the incorrect treatment, mortality rates of 42–100% greater than patients receiving the correct treatment [18,20]. Additionally, β-lactams could promote horizontal gene transfer of virulence factors [21] and could be responsible for the spread of ESBL genes. As a precaution, most first-line β-lactams are ruled out if ESBL-producing pathogens are detected, even for ESBL-producing pathogens that appear to be sensitive to a particular β-lactam [22–25]. This is done largely out of concern for complicating drug responses that have been observed *in vitro*, such as the inoculum effect, a phenomenon in which the minimum inhibitory concentration (MIC) of an antibiotic increases as the bacterial density increases [24,26–30].

With first-line β-lactams ruled out, second-line antibiotics, such as carbapenems, fluoroquinolones, β-lactam/β-lactamase inhibitor combinations, glycopeptides, and cephamycins, are typically administered [31]. Although this practice is based on a valid concern, it has limitations. Specifically, second-line antibiotics are associated with higher costs and more adverse effects [32–37]. Additionally, the more frequently bacteria are exposed to second-line antibiotics, the faster the pathogens are likely to develop resistance to our last resort antibiotics [2,5]. Given the dearth of new antibiotics entering the market and the limited number of effective antibiotics already available, we cannot afford to disregard potentially effective antibiotics.

First-line β-lactams could represent a missed opportunity for treating pathogens producing moderate levels of ESBLs. Individual bacteria that produce moderate levels of ESBL can remain sensitive to the antibiotic due to insufficient production or activity of ESBL; however, if enough bacteria are present, then the population’s collective ESBL concentration will be sufficient to render the population resistant to the antibiotic [38,39]. In other words, a low density population of moderate ESBL producers would lyse entirely because its collective ESBL concentration would be insufficient to inactivate the β-lactam, while a high density population would only experience partial lysis before its collective ESBL concentration can inactivate the β-lactam and promote the recovery of the surviving bacteria. This collective population recovery is time dependent [40]. Shortly after the antibiotic is first applied, the population will be reduced due to lysis and appear susceptible because it will not have yet benefited from the activity of ESBLs. Ideally, a treatment could pinpoint the time window when the most lysis has occurred and the least benefit has been experienced.

Extensive studies have been carried out to devise methods to optimize treatment efficacy of antibiotics by changing the dosing period and amplitude. These studies typically examine which metric(s) can capture the pharmacokinetic/pharmacodynamics (PK/PD) of an antibiotic and be used to predict antibiotic efficacy [41–44]. Current metrics adopted in the clinical setting, such as the MIC, do not account for the time course of antimicrobial activity and are not sufficiently predictive of treatment efficacy [22,45–47].

Therefore, there is a need for a simple metric that characterizes this pathogen-antibiotic interaction that can be easily measured and used to design dosing protocols that will effectively clear an infection. Here, we use quantitative modeling to demonstrate a strategy for customizing regimens for a particular bacteria and antibiotic combination without needing to know the full mechanistic basis for the bacteria-antibiotic interaction. Specifically, we focus on optimizing a dosing protocol to enable β-lactams to effectively treat a moderate ESBL-producing pathogen. To help guide the design of effective protocols, we develop a metric, the recovery time, which is easy to measure and quantifies the pathogen-antibiotic interaction. Even though we assumed specific molecular mechanisms underlying this collective antibiotic response, our model illustrates that the predictive power of the recovery time is maintained for different specific molecular mechanisms and for different initial conditions. Through optimized antibiotic regimens, our strategy could extend the use of first-line antibiotics, improve treatment outcome, and preserve last-resort antibiotics.

## Results

### Model development and characterization

**Fig 1:**
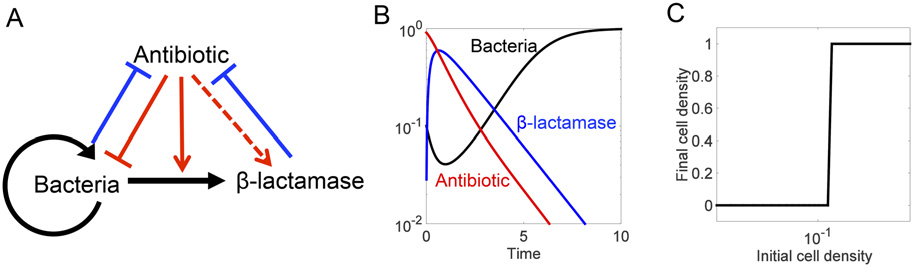
Mechanism and dynamics of antibiotic-mediated death. **(A) Antibiotic-mediated death**. Black represents bacterial actions, blue represents Bla actions, and red represents antibiotic actions. Arrows denote induction or activation; T-lines indicate inhibition; the dashed arrow represents the ability for the model to simulate inducible or constitutive Bla production. **(B) Typical time courses of bacterial density, antibiotic, and Bla after one dose of antibiotic treatment**. The antibiotic can cause cell lysis, which triggers the release of Bla into the environment. Sufficient degradation of the antibiotic by the Bla allows the surviving bacteria to recover. **(C) Collective tolerance**. A bacterial population can only recover from an antibiotic dose if enough bacteria are present for sufficient Bla to be produced.

We developed a kinetic model comprising a set of ordinary differential equations (ODEs) to capture the population dynamics of collectively tolerant, ESBL-producing bacteria being treated by a β-lactam (S1 Text) [40]. We further nondimensionalized the model to facilitate analysis. In this model, introduction of the antibiotic inhibits bacterial growth and causes lysis. β-lactamase (Bla) is naturally found in the periplasm of Gram-negative bacteria, where it can benefit the host bacterium by hydrolyzing the β-lactams that diffuse into the periplasm [48]. However, moderate amounts of periplasmic Bla are insufficient to protect a bacterium from high concentrations of antibiotic [38,49]. Conversely, sufficient amounts of Bla can accumulate to protect a population if enough bacteria are initially present. With a dense enough population, the collective intracellular and extracellular Bla, due to lysis or leaky secretion [50], will be sufficient to degrade the antibiotic to a sublethal concentration before all cells are eliminated (Fig. 1A). Thus, the survival of the population depends on establishing a collective antibiotic tolerance (CAT)[30]. In general, Bla expression can be constitutive or inducible by the antibiotic [51–54]. Here, we focus on constitutive Bla expression, which is most clinically relevant to the pathogens that express plasmid-mediated ESBLs [39,55]. However, our conclusions also apply to the case where Bla expression is inducible. They will likely apply to bacterial responses to other antibiotics if the antibiotic causes an initial decline in the population density by killing a subpopulation of cells and the population can recover when the antibiotic is subsequently degraded by an enzyme produced by the cells (whether or not the enzyme is released into the culture).

Using physiologically relevant parameters, our model generates PK/PD profiles that are characteristic of Bla-mediated CAT. Starting from a sufficiently high initial density, the population exhibits an initial decline upon antibiotic treatment, followed by eventual recovery due to intrinsic and Bla-mediated degradation of the antibiotic (Fig. 1B). Sufficient time is needed to observe this apparent drug tolerance. If examined shortly after antibiotic treatment, the population will have just experienced significant lysis and will appear susceptible because the effects of Bla have not yet been fully recognized.

For a fixed initial antibiotic concentration, the model predicts a switch-like dependence of population survival over the initial population density: the population can only survive if starting at a sufficiently high density (Fig. 1C). If too few bacteria are present, the total expression of Bla from the entire population is insufficient to degrade the antibiotic fast enough to allow the population to recover. If enough bacteria are present, however, the population can endure the initial crash in density for a longer period. As such, some bacteria remain when the antibiotic concentration decreases sufficiently, due to Bla-mediated degradation, to allow the population to recover. The density-dependent survival of the population is the defining feature of the inoculum effect [28,56].

### Recovery time as a metric to quantify bacterial response

Our results illustrate the defining features of a CAT bacterial response involving antibiotic-triggered death. In particular, the population will appear resistant when its initial density is sufficiently high and it is given enough time to recover. These features form the basis for the preemptive practice of disregarding β-lactams when an ESBL-pathogen is identified. However, our model also indicates that the population is sensitive when its initial density is sufficiently low or when it is examined in a short time window. Given these properties, we reason that optimal antibiotic dosing may remain effective in eliminating bacteria. If so, an immediate next question is how to best design the treatment protocol.

This task would be straightforward if we could determine the specific molecular mechanisms and defining parameters for each pathogen-antibiotic pair: under such a scenario, we could in theory use a model specific to the pair to examine efficacy of different dosing protocols. This is impractical, however, as many ESBL pathogens are poorly characterized at the molecular level and there are many different ESBL enzymes [57]. A more practical option would be to identify an easy-to-measure, *lumped metric* based on a bacterial population’s response to a single dose of antibiotic that will allow us to reliably predict its response to periodic antibiotic treatment without needing to know the underlying molecular-level parameters.

**Fig 2:**
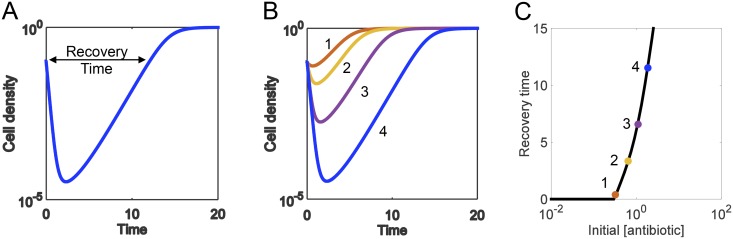
Defining the recovery time. **(A) Recovery time defined**. The recovery time is the time it takes for a population to return to its starting density after being exposed to a dose of antibiotic. **(B) Each antibiotic concentration has a recovery time**. To capture both the time- and concentration-dependent relationship between an antibiotic and the bacterial population, the recovery time is measured for a range of an antibiotic’s concentrations. (Increasing numbers labeling the curves correspond to increasing concentrations of antibiotic: 1 = weakest, 4 = strongest). **(C) The recovery time curve links pathogen to an antibiotic**. The recovery times for a range of antibiotic concentrations produce an informative curve that represents the interaction between a pathogen and a particular antibiotic.

A typical metric to quantify efficacy of an antibiotic is the minimum inhibitory concentration (MIC), which can be measured by disk diffusion and microbroth dilution methods after a certain duration of antibiotic treatment [58]. However, the MIC measured at a particular time point does not capture the rich temporal dynamics of bacterial responses due to antibiotic-triggered death. Instead, we propose to use another lumped metric: the recovery time; specifically, this defines the time it takes a population to return to its initial density after being exposed to a dose of antibiotic (Fig. 2). By definition, the recovery time captures the dominant dynamic features of bacterial temporal response. As such, it may be a more predictive metric for the long-term outcome of periodic antibiotic treatment.

### Predictive power of the recovery time for injection-based protocols

**Fig 3:**
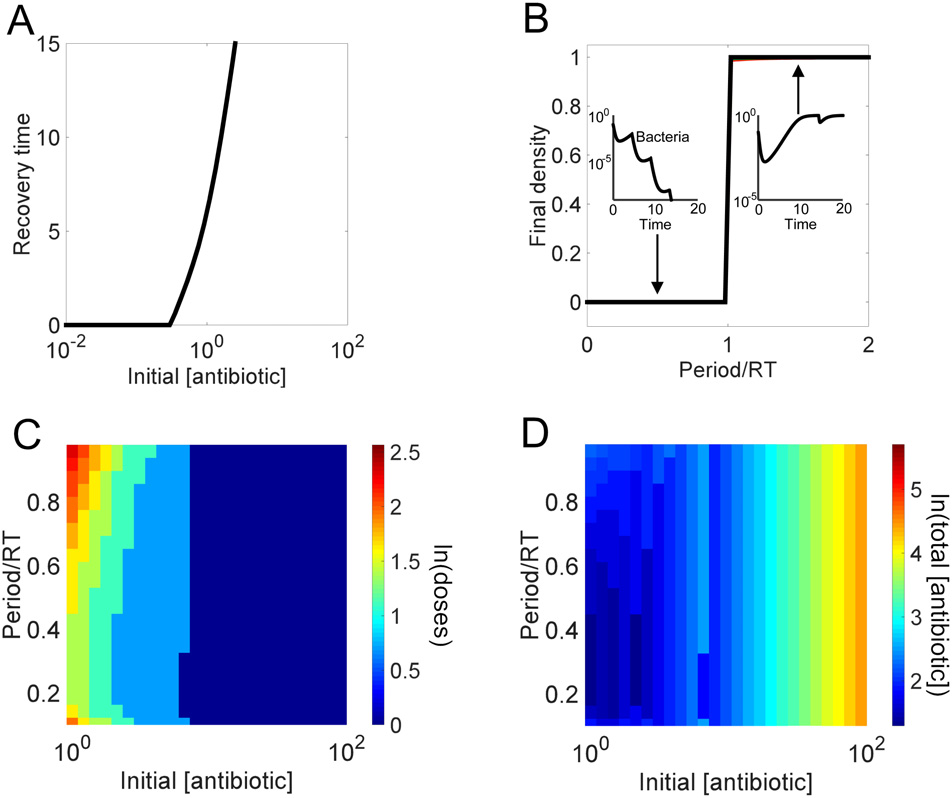
Recovery time guides design of effective injection based regimen. **(A) Dependence of the recovery time on the initial antibiotic concentration**. If the initial antibiotic concentration is too low, then the population will not be affected and its recovery time will be zero. However, after the initial antibiotic concentration is high enough, increasing the concentration results in an increase in the time it takes for a population to recover from a single dose. **(B) Predictive power of recovery time for the outcome of long-term periodic antibiotic dosing**. For each antibiotic concentration-period combination, we calculate the final population density after 100 antibiotic doses. Subplots demonstrate the outcomes for the first couple of doses of regimens using periods less than one recovery time (bacteria final density is below the defined threshold of 10^-10^) versus regimens using periods greater than one recovery time (bacteria final density returns to carrying capacity). **(C) Dependence of treatment efficiency on the antibiotic concentration and the dosing period**. Each combination using an antibiotic concentration with a recovery time > 0 (*a*_0_ > 0.5) and any period less than 1 recovery time can eventually eliminate the population. Different combinations will reduce the population density to a pre-defined threshold (10^-10^) with varying efficiency: the combination is more efficient if fewer doses are needed to reach the threshold. *a*_0_ < 0.5 could not clear the infection in 100 doses. **(D) Dependence of total antibiotic usage on the antibiotic concentration and dosing period**. The total usage is calculated as the antibiotic concentration multiplied by number of doses needed to reduce population density to a predefined threshold.

We first tested the predictive power of the recovery time in injection-based dosing protocols. With the base-parameter set, our model predicts a monotonic dependence of the recovery time on the antibiotic concentrations for single-dose treatment (Fig. 3A). Once the initial antibiotic concentration is high enough to cause cell lysis (*a*_0_ > 0.5), the recovery time increases exponentially with the initial antibiotic concentration until the antibiotic concentration is too high (*a*_*0*_ > 10) and the recovery time becomes infinite. This dependence is an intrinsic property of antibiotic-mediated lysis. Under low concentrations of antibiotic (0.5 < *a*_0_ < 10), the recovery time is primarily determined by how fast the antibiotic is degraded by Bla. Under increasing concentrations of antibiotic (*a*_0_ > 10), the rate of antibiotic degradation is essentially saturated (limited by the population size and the constant production rate of Bla) and the recovery time is primarily determined by the lysis rate. β-lactams’ killing rate is time-, not dose-, dependent and is reflected in the model’s lysis rate’s non-linear dependence on the antibiotic concentration (Hill coefficient = 3) [59]. Once the antibiotic concentration is high enough, further increasing the concentration does not increase the lysis rate.

As noted above, the recovery time could represent a simple, yet reliable, metric in predicting outcomes from periodic treatment. To test this notion, we examined the consequence of periodic dosing of varying antibiotic concentrations. For each concentration, we varied the dosing periods from 0.1 to 2 times the corresponding recovery time, and obtained the final population density after 100 doses. Our modeling results confirmed the predictive power of the recovery time: as long as the initial antibiotic concentration is sufficiently high to cause significant initial lysis, the population will reach a high final density if the period is greater than the recovery time; the population goes extinct otherwise (Fig. 3B).

Of the regimens leading to eventual population extinction (period < recovery time), different combinations of antibiotic concentrations and dosing periods eliminate a population with varying efficacy. To quantify this efficacy, we calculated the minimum number of doses necessary to reduce the population density to below 10^-10^ (Fig. 3C). The resulting landscape shows a strong dependence on antibiotic concentration and the corresponding recovery time. When the antibiotic concentration is too low and the recovery time is close to 0, the number of doses required to clear the infection is very large, regardless of the dosing frequency. When the antibiotic concentration is very high and the corresponding recovery times approach infinite, then the number of doses is very low. However, there is an intermediate range of antibiotics with intermediate recovery times that show variation in the number of doses necessary to clear the infection. Concentrations producing the longer recovery times require fewer doses because they can reduce the bacterial density more severely than concentrations with shorter recovery times.

For intermediate antibiotic concentrations (1 < *a*_*0*_ < 10) to be most effective, the model suggests they should be delivered at low-to-intermediate period lengths (period = 20–50% recovery time) at which the population is most vulnerable. At the end of each period, the bacteria are still lysing, have almost reached minimum density, but have not yet experienced the benefits of Bla. At this point, the antibiotic has not been completely removed; thus the population will be subjected to a slightly higher concentration of antibiotic at each additional dose. If the antibiotic is delivered too frequently, the accumulated antibiotic increases the rate of lysis, thus causing higher amounts of Bla to be released, ultimately leading to the faster removal of the antibiotic. However, Bla cannot fully degrade the antibiotic before the next dose is added and the population quickly dies off. Although the population is cleared, a higher number of doses is necessary because the degree of lysis per dose is not maximized. In other words, subsequent doses are applied before the full extent of lysis from the previous dose is observed. However, if the antibiotic is delivered too infrequently, then the population will have the chance to recover between doses. Once again, these conditions do not maximize the degree of lysis per dose and more doses are necessary to achieve the same amount of population decrease associated with doses applied more frequently.

A final aspect to consider when designing a regimen is the total amount of antibiotic delivered (Fig. 3D). Although some of the model’s regimens using higher concentrations of antibiotic (*a*_0_ > 10) are associated with fewer doses, they have the highest net antibiotic concentration. These concentrations may not be optimal, due to potential adverse effects associated with using excessive amounts of antibiotic, such as the destruction of the normal microbial flora, interference with the immune response, increased nephrotoxicity, and selection for antibiotic resistant mutants [32,60–63]. Also, efficient use of antibiotics can help reduce treatment cost [14,35]. Using dose number and total antibiotic delivered, an effective and realistic regimen can be designed by minimizing the number of doses, the delivery frequency, and the total antibiotic delivered.

We note that the predictive power of the recovery time is maintained for low or moderate inoculum sizes. In particular, our modeling demonstrates that a multi-dose regimen will clear a population if the time between doses is less than one recovery time, regardless of effective antibiotic concentration and inoculum size (S1 Fig). Similar to the base case, the regimen can be optimized to have the fewest doses and the lowest net antibiotic concentration delivered by selecting the lowest concentration of antibiotic associated with the longest recovery time.

Additionally, the predictive power of the recovery time is maintained for an antibiotic with dose-dependent killing (Hill coefficient = 1) or an antibiotic with time-dependent killing (Hill coefficient = 10): a multi-dose regimen will clear a population if the time between doses is less than one recovery time, regardless of effective antibiotic concentration and degree of antibiotic-mediated killing (S2 Fig).

The predictive power of the recovery time can be applied to bacteria with varying rates of Bla synthesis and accumulation as long as the antibiotic concentration applied has an effective recovery time (S3 Fig A-T). When the bacteria are producing and accumulating Bla at a very fast rate (S3 Fig P-T), most individual bacteria can sufficiently protect themselves (CAT is no longer necessary) and the population experiences little or no decline in density. Consequentially, the model predicts that effective treatment protocols would shift to higher antibiotic concentrations capable of inducing significant lysis in more resistant bacteria.

The predictive power is upheld as long as the recovery times associated with subsequent doses are sufficiently similar to the original recovery time measured from a single dose. Recovery times of subsequent doses depend on two main factors: the activity of Bla in the environment and the concentration of antibiotic. On one hand, if there is insufficient time for Bla to degrade between doses, then it will compound with each dose until the population is being protected by higher concentrations of Bla, relative to when the first dose was administered. As a result, the increasing pool of Bla will degrade the antibiotic faster, the recovery time of subsequent doses will decrease, and the population can recover when dosed at period lengths less than the original recovery time (S4 Fig A-B). This would happen in scenarios where the antibiotic concentration applied is insufficient to counterbalance the Bla that is either expressed at high levels or has an increased rate for hydrolyzing an antibiotic. The loss of predictive power in this case can be avoided by using a sufficiently strong antibiotic concentration. On the other hand, if there is insufficient Bla to degrade the antibiotic between doses, then the antibiotic will compound with each dose until the population is being exposed to higher concentrations of antibiotic, relative to when the first dose was administered. As a result, the increasing concentration of antibiotic will kill more cells, the recovery time of subsequent doses will increase, and the population will not be able to recover when dosed with period lengths equal to the original recovery time (S4 Fig C-D).

### Predictive power of recovery time for intravenous-drip protocols

**Fig 4:**
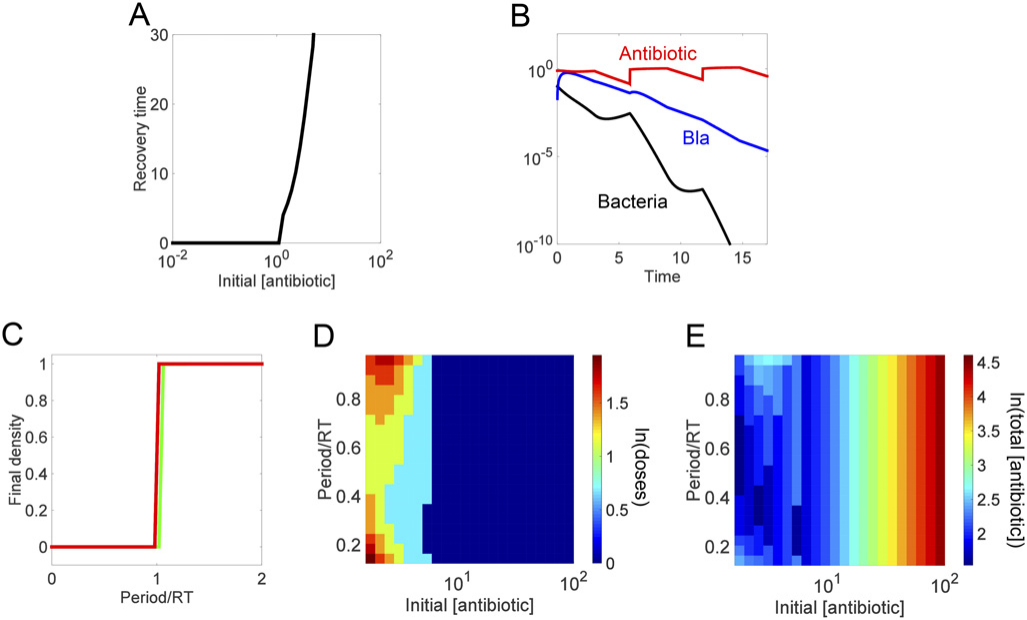
Recovery time guides design of effective intravenous drip based regimen. **(A) Dependence of the recovery time on the antibiotic concentration during IV**. We maintain each concentration for a fixed duration (time = 3) and then calculate the corresponding recovery time. **(B) Time course of an IV-drip regimen**. The antibiotic was delivered for a fixed duration until the bacteria density dropped below a pre-defined threshold (10^-10^). **(C) Predictive power of recovery time for the outcome of long-term periodic antibiotic dosing**. For each antibiotic concentration-period combination, we calculate the final population density after applying 100 antibiotic doses. **(D) Dependence of treatment efficiency on the antibiotic concentration and the dosing period**. The efficacy is determined in the same manner as in Fig. 3. **(E) Dependence of total antibiotic usage on the antibiotic concentration and the dosing period**. The total usage is calculated as in Fig. 3.

Many antibiotics, such as β-lactams, are most effective when applied continuously for long periods of time [64,65]. Thus, we also modeled the predictive power of the recovery time in intravenous (IV)-drip based protocols, where a set concentration of antibiotic is delivered over a set duration during each dosing period. Here, we delivered the antibiotic dose over three time units and measured the corresponding recovery time (Fig. 4A). Similar to the injection recovery times, the IV-drip recovery times increase monotonically as the concentration of the dose increases, more Bla is required to remove the antibiotic, and more of the population lyses. In contrast, the IV-drip therapy has a narrower range of intermediate antibiotics with 0 < recovery time < 100. Some of the lower concentrations that are effective for injection treatment (0.5 < *a*_*0*_ < 1) are ineffective for IV drip treatment because the dose is too weak when delivered over a longer period of time. However, when the dose concentration is sufficiently high, the IV-drip recovery time is longer than the injection recovery time because the IV-drip is exposing the bacteria to a higher concentration for a longer period of time (Fig. 4B).

Again, we use the recovery time from a single IV dose to establish the range of dosing frequencies able to eliminate the bacterial population. At each dosing concentration (for a fixed time duration of 3), we applied 100 doses of the antibiotic at periods ranging from the infusion duration (*τ* = 3) to 2 times the corresponding recovery time and calculated the resulting final bacterial density. The model shows that the predictive power of the recovery time is maintained when the antibiotic dosing concentration is sufficiently large with a long enough recovery time (*a*_*0*_ > 1.5): a multi-IV-dose regimen will eventually eliminate the population if the dosing period is less than one recovery time, regardless of effective antibiotic concentration (Fig. 4C). There is slight deviation from this for *a*_*0*_ < 1.5 due to the corresponding recovery times being too short for the Bla to be reduced to a baseline concentration before the next round of lysis and Bla release occurs. As a result, periods less than one recovery time could fail to eradicate the infection because the Bla concentration compounds with each subsequent dose, the antibiotic is degraded more quickly, fewer cells lyse, and the population can recover (S4 Fig).

Similar to the injection based therapy, the IV-drip reduced a population constitutively producing high concentrations of Bla as long as the period was less than one recovery time and the initial antibiotic concentration was sufficiently high to cause significant initial decline (S3 Fig U-Y). However, the IV-drip protocols retained a larger range of effective antibiotic concentrations than the injection based protocols. This robustness is due to the antibiotic concentration continuously being replenished from the IV-drip. If a high enough concentration is maintained for sufficient time, the population’s Bla concentration will not be able to remove the antibiotic fast enough to prevent lysis and the population will decrease with each subsequent round of IV-drip infusion. Thus, these results suggest that IV-drip based regimens could serve as a platform for effectively applying first-class β-lactams to clear constitutive producers of high levels of ESBLs.

We next evaluated the efficacy of each effective concentration-period combination by calculating the minimum number of doses necessary to reduce the population density to below 10^-10^ (Fig. 4D). Relative to the injection protocol, the IV-drip therapy has a narrower region of intermediate dose numbers, reflecting the narrow region of intermediate recovery times. Similarly to the injection based regimens, the intermediate antibiotic concentrations (1 < *a*_*0*_ < 5) require the least doses when delivered at low-to-intermediate period lengths (period = 20–60% recovery time) because that is when the population is most vulnerable. Again, the initial antibiotic concentrations too low to have a recovery time (*a*_*0*_ < 1) do not clear the infection, regardless of the dosing interval or number of doses applied. The concentrations with an infinite recovery time (*a*_*0*_ > 5) require only a single dose and thus the dosing frequency does not matter.

Although the number of doses necessary to clear an infection might be the same for a range of antibiotic concentrations and periods, the least amount of total antibiotic is needed for intermediate antibiotic concentrations applied at 20–60% of the associated recovery time (Fig. 4E).

### Predictive power of the recovery time in mixed populations

A bacterial population often consists of phenotypically or genetically heterogeneous subpopulations[66,67]. For instance, different cells may express different levels of Bla, have different growth rates, or exhibit different sensitivities to the same antibiotic. This heterogeneity could compromise the predictive power of the recovery time. To examine this notion, we extended our injection-based model to account for two cases, each dealing with a mixture of two subpopulations (S1 Text). In one case, one subpopulation grows much more slowly and exhibits much greater tolerance to the antibiotic. In the other, two subpopulations display different degrees of collective antibiotic tolerance.

#### Case I: A mixture consisting of normal cells and persister cells

The first case accounts for the impact of persisters. Persisters are non- or slow-growing bacteria that are genetically identical to susceptible cells, but are highly tolerant to antibiotics [68–73]. Because persisters are generated at low frequencies and can only start to reestablish the population upon removal of the antibiotic [71,74,75], the overall dynamics of persisters will happen at a much slower time scale than for normal cells. Here, we assume persisters form a small fraction of the initial population (< 1%), transition to and from normal cell phenotypes when the antibiotic concentration is low enough, and grow and lyse at rates 100 to 1000 times slower than normal cells [70,72]. When antibiotic concentrations are low (*a*_0_ < 0.3), the recovery time is 0, regardless of the presence of persisters. As antibiotic concentration starts to increase (0.3 < *a*_0_ < 26), the population of normal cells starts to undergo the population crash and recovery while the persisters grow and die very slowly (S5 Fig A,F). Once the antibiotic has been sufficiently degraded, then the persisters start being generated from and returning to the normal population. This ability to transition between phenotypes is what allows for a population containing persisters to recover under antibiotic concentrations (*a*_0_ > 26) that would normally kill a population without persisters (S5 Fig B,G). The predictive power of the recovery time is upheld for the population with persisters growing and dying 100fold more slowly, but not 1000fold more slowly (S5 Fig C,H). Although the normal population is reduced below the threshold after a few doses, the persisters’ net death rate determines the duration that the antibiotics need to be applied. The persisters growing and lysing 100fold more slowly than normal cells have a death rate high enough to reduce the persisters to 0 before the 100 doses of antibiotic have been applied at periods less than 1 recovery time (S5 Fig D). The fewest doses are needed for regimens applying higher concentrations of antibiotic (*a*_0_ > 26) at period lengths > 0.3 recovery time. The persisters growing and lysing 1000fold more slowly are still present at a low density at the end of most of the different regimens tested. Here, the successful regimens are those delivering high concentrations of antibiotic (*a*_0_ > 10) with long period lengths (period > 0.65 recovery time) (S5 Fig I). Similar to the previous models, the persister model shows that regimens applying higher dose concentrations deliver a higher overall concentration of antibiotic (S5 Fig E,J). These results suggest that, while the recovery time can help optimize regimens for relatively slow- growing and lysing persisters, the power is lost for extremely slow- or non- growing persisters. This further confirms that if our model’s central assumption, that the population is collectively antibiotic tolerant, is violated, then the predictive power of the recovery time will diminish.

#### Case II: A mixture consisting of two distinct subpopulations that are both sensitive to the antibiotic

Here, the divergence between the two subpopulations is much less than that between normal and persister cells. The more resistant subpopulation has thresholds of growth inhibition and lysis five times greater than the moderately resistant subpopulation. As a result, the more resistant subpopulation does not lyse as much and recovers faster than the moderately resistant subpopulation (S6 Fig A,F). If the subpopulations can switch between states[76,77], the more resistant subpopulation can re-establish the moderately resistant subpopulation once the antibiotic concentration is sufficiently low. Otherwise, the moderately resistant subpopulation will not recover. Because both subpopulations still undergo the process of lysing before recovering, the dosing protocol based on recovery time is still applicable (S6 Fig B,G). For 0.3 < *a*_0_ < 2.3, both subpopulations recover at similar rates, thus the population as a whole recovers quickly. For 2.3 < *a*_0_ < 26, the more resistant subpopulation recovers faster and determines the population level recovery time. For *a*_0_ > 26 neither subpopulation can recover. The predictive power is upheld for this mixed population: a multi-dose regimen will clear a population if the time between doses is less than one recovery time, regardless of effective antibiotic concentration and degree of antibiotic-mediated killing (S6 Fig C,H). Similar to other scenarios, the dosing number and total antibiotic delivered can be determined and optimized, based on the antibiotic concentration and period length (S6 Fig D-E, I-J). These results suggest that the recovery time could be used to optimize treatments for heterogeneous populations with more than two subpopulations, as long as the phenotypic difference between these subpopulations is not drastic.

## Discussion

Most antibiotic regimens are based on empirical observations of how bacterial infections responded to an antibiotic [32,78,79]. However, these regimens may be suboptimal both because they were not initially designed to handle resistant bacteria and because the current diagnostic assays cannot accurately predict how resistant pathogens will respond to them. It is critical that we develop a new strategy for using the existing antibiotics more effectively or our medical care will return to a state equivalent to that of a pre-antibiotic era. Ideally, the new strategies would be based on the molecular mechanisms underlying antibiotic resistance. However, this is impractical, given that many pathogens’ resistance mechanisms have not been characterized and they evolve rapidly. To this end, we propose using the recovery time as a lumped metric that can characterize a pathogen’s response to an antibiotic without requiring knowledge of the underlying mechanism.

We used a kinetic model to test the ability of the recovery time to predict ESBL-producing pathogens’ responses to periodic dosing of β-lactams. Our simulation results suggest that the recovery time of a single dose can be used to design optimal multi-dose regimens for multiple delivery methods, including injections and continuous IV drip, various inoculum sizes, bacteria with a range of Bla production levels, and certain heterogeneous populations. Optimal dosing regimens for treating Bla-producing bacteria with a β-lactam would apply intermediate concentrations of antibiotic that have long recovery times at time intervals corresponding with when the bacterial density has been minimized. Furthermore, our modeling results suggest that regimens using lower, yet still lethal, concentrations of antibiotic can be as effective as regimens using higher concentrations. Reducing the amount of antibiotic the host is exposed to may be important to minimize the perturbation of the host’s microbiota and other defense mechanisms [32,60–62,80], which could have long-lasting detrimental effects. Also, under certain conditions, a higher concentration of antibiotic can lead to selection of more resistant subpopulation of bacterial pathogens [81].

Although this model considers the population level response to an antibiotic, there is a significant amount of gene-expression noise at the single cell level [66,67]. If an antibiotic were applied such that the population would have the chance to recover between doses, then the antibiotic would select for the bacteria expressing higher levels of resistance genes (as demonstrated in S6 Fig F). Ultimately, this would direct the evolution of the population towards an inherently more resistant infection than before the antibiotic treatment was applied. Our proposed method would minimize this problem by delivering subsequent doses of antibiotic before a more resistant population grew to a significant density.

**Fig 5:**
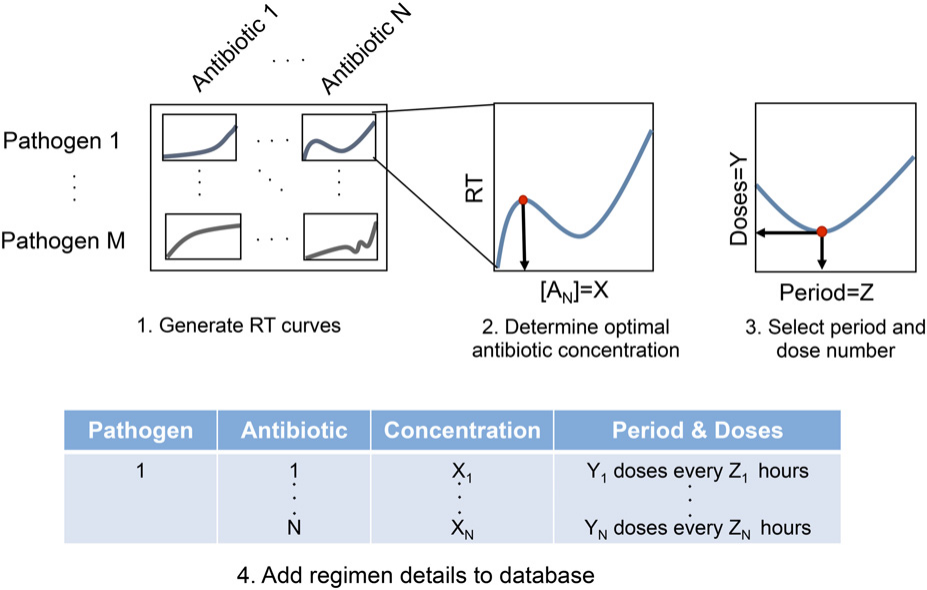
Potential use of recovery time to guide clinical practice. A critical step entails the construction of a comprehensive database of the recovery time curves of various pathogens under different antibiotics. Based on the recovery time curve, the optimal antibiotic concentration (X), dose number (Y), and period length (Z) can be calculated for each pathogen-antibiotic combination and entered into a database. Given this database and a proper diagnosis of a pathogen, one can readily identify the most effective treatment protocol.

The recovery time of a pathogen under a single dose of antibiotic is a metric that is easy to measure and could guide the choice of an appropriate multi-dose antibiotic regimen for a wide range of infections. Measurements of the recovery time can be carried out in high resolution using commercially available microplate readers [82]. A critical step entails the construction of a comprehensive recovery time database for various pathogens under different antibiotics (Fig. 5). When a new bacterial pathogen is identified, its recovery times to a range of antibiotic concentrations will be recorded *in vitro* for different starting densities. Based on these measurements, regimens with varied concentrations and period lengths will be tested for different inoculum sizes. From these results, the period length, dose number, and antibiotic concentration can be optimized for a particular pathogen *in vitro*. Before entering this information into the database, the PK/PD of the particular antibiotic will be necessary to determine the concentration of antibiotic that should be delivered such that the concentration at the site of infection matches the concentration selected from the *in vitro* experiments. Given this database, a proper diagnosis of a pathogen, and an estimate of the severity of the infection (e.g. inoculum size), one can readily identify the scenarios in which first- and second-line antibiotics may still be applied and chose the most effective treatment protocol. Whenever a new pathogen arises, it can be evaluated and added to the library.

The ability to predict the outcome of a multi-dose treatment without knowing the underlying resistant mechanism would remove the uncertainty that prevents clinicians from using first-line β-lactams when an ESBL-producing pathogen is detected. Given ESBL-producing bacteria’s prevalence [19,83–85], our proposed strategy could help to minimize the rate at which these bacteria develop resistance to more extreme antibiotics by ensuring that we do not overlook effective first-line antibiotics before moving on to more extreme antibiotics.

## Methods

The interaction between a β-lactam and a bacterial population expressing Bla can be simplified to the interactions between three main components: population density (*n*), antibiotic concentration (*a*), and Bla concentration (*b*). Our base model consists of the following ordinary differential equations:

$$\frac{dn}{d\tau }=\left(g-l\right)n$$ (1)

$$\frac{db_{out}}{d\tau }=lb_{in}^{*}-\gamma_{2}b_{out}-\kappa_{IV}\left(\tau \right)b_{out}$$ (2)

$$\frac{da\;}{d\tau }=\kappa_{IV}\left(\tau \right)a_{inject}-\left(b_{out}+\alpha b_{in}^{*}\right)\left(\frac{a}{1+a}\right)-\gamma_{3}a-\kappa_{IV}\left(\tau \right)a$$ (3)

$$g=\left(1-n\right)\left(\frac{\sigma_{1}}{\sigma_{1}+a}\right)$$ (4)

$$l=\gamma_{1}\left(\frac{a^{H}}{\sigma_{2}^{H}+a^{H}}\right)\left(\frac{\sigma_{4}}{\sigma_{4}+b_{in}}\right)$$ (5)

$$b_{in}=\kappa \left(\frac{r}{g+\gamma_{4}}\right)$$ (6)

$$b_{in}^{*}=\beta nb_{in}$$ (7)

$$r=\frac{a}{\sigma_{3}+a}$$ (8)

where *g* and *l* represent bacteria growth and lysis, respectively. Initial conditions of *n*(0) = 0.1, *b*(0) = 0, and *a*(0) = 0.01–100 were used for all simulation results, except for S2 Fig where *n*(0) = 0.01 or 0.001. The rest of the parameters are defined in a table in S1 Text. See S1 Text for further details of the model development and for the extended models that account for heterogeneous populations. Minor modifications are introduced to account for the IV drip protocol or dynamics of a mixture consisting of two subpopulations (S1 Text).

## Supporting Information

S1 Text Model development. The equations, nondimensionalizing terms, and parameter values are detailed here for the different models.
(DOCX)

S1 Fig The effect of initial cell density on the predictive powers of recovery time. **(A,F) Time courses** for populations with initial densities that are 10x and 100x smaller than the base model. **(B,G) Recovery time curves** for populations with initial densities that are 10x and 100x smaller than the base model. **(C,H) Final density depends on dosing frequency**. Despite the lower initial densities, both models followed the trend where periods less than one recovery time eliminate the population as long as the initial antibiotic concentration is sufficiently high to cause significant initial decline. This indicates that the recovery time is a viable tool for predicting treatment outcomes for a range of population sizes. **(D,I) Dose number necessary to reduce a population below critical threshold depends on antibiotic concentration and period length**. The fewest number of doses corresponds to the antibiotic concentrations with the longest recovery times; however, intermediate concentrations can be effective when applied at low to intermediate period lengths. **(E,J) Total antibiotic concentration delivered depends on single dose concentration**. The regimens applying doses of lower concentrations of effective antibiotic will eliminate the population just as effectively as the regimens using high concentrations, but with less total antibiotic.
(PDF)

S2 Fig The effect of the Hill coefficient on the predictive powers of recovery time. **(A,C) Recovery time depends less on antibiotic concentration if the Hill coefficient (H) is high enough**. When H = 1, the recovery time is dose dependent, increasing as the antibiotic concentration increases. When H = 10, the recovery time quickly transitions from being 0 to infinite (the population has been wiped out). Once past the threshold, increasing the antibiotic concentration will not continue to increase the recovery time. **(B,D) Final density depends on dosing frequency**. Despite the different Hill coefficients, both models followed the trend where periods less than one recovery time eliminate the population as long as the initial antibiotic concentration is sufficiently high to cause significant initial decline. This indicates that the recovery time is a viable tool for predicting treatment outcomes for a range of antibiotics with different modes of killing (time vs. dose dependent).
(PDF)

S3 Fig The effect of Bla production levels on the predictive powers of recovery time. **(A-E) No Bla production. (A) Time course**. The recovery of bacteria that do not produce Bla depends on the intrinsic removal of the antibiotic caused by natural degradation and turnover by the body’s fluid. **(B) Recovery time curve**. Because the bacteria still undergo the process of lysing before recovering, the dosing protocol based on recovery time is still applicable. Without the production of Bla to aid in the recovery of the population, the recovery time is longer and monotonically dependent on the antibiotic concentration. **(C) Final density**. Protocols using periods of less than one recovery time are effective at eliminating the population, regardless of antibiotic concentration. **(D) Dose number**. For each antibiotic concentration and period combination, the corresponding number of doses necessary to eliminate the population was calculated. The regimens using antibiotics associated with longer recovery times require the fewest doses; however, low concentrations of antibiotic (*a*_0_ ≈ 1) can be effective if applied at period lengths of 0.10–0.50 period/RT. **(E) Total antibiotic delivered**. Despite many different regimens requiring the same number of doses to clear an infection, the regimens could be differentiated by the total amount of antibiotic delivered. When comparing regimens with the same number of doses, the amount of antibiotics delivered decreases as the dose concentration decreases. **(F-J) Low inducible Bla production. (F) Time course**. The population recovers faster than a population that does not produce Bla because it generates sufficient Bla to degrade the antibiotic; however, the recovery time is slower than a population that constitutively produces Bla because more bacteria lyse before sufficient Bla accumulates to effectively remove the antibiotic. **(G) Recovery time**. The recovery time increases as the initial antibiotic concentration increases. **(H) Final density**. Protocols using periods of less than one recovery time are effective at clearing an infection, regardless of antibiotic concentration. **(I) Dose number**. A larger range of antibiotic concentrations require the lowest number of doses because the population is more sensitive to lower antibiotic concentrations, relative to constitutive producers. **(J) Total antibiotic delivered**. Despite the higher number of doses, the least amount of antibiotic is delivered for regimens using lower antibiotic concentrations. **(K-P) High Inducible Bla Production. (K) Time course**. Increased Bla production results in less lysis, faster antibiotic degradation, and, consequently, a faster population recovery. **(L) Recovery time curve**. The non-monotonic dependence reflects the generation of the Eagle effect: a higher antibiotic concentration can generate faster population response. **(M) Final density**. Protocols using periods of less than one recovery time are effective at clearing an infection, regardless of antibiotic concentration. **(N) Dose number**. The dose number is highly complex in terms of the dependence on the initial antibiotic concentration and period length. The dependence on the recovery time, however, appears simpler: the fewest number of doses is needed for antibiotic concentrations with the longest recovery times that are delivered at intermediate-to-long dosing frequencies. Concentrations producing the longer recovery times require fewer doses because they can reduce the bacterial density more severely than concentrations with shorter recovery times. **(O) Total antibiotic delivered**. Despite the higher dose numbers, the least antibiotic is delivered for regimens applying low concentrations of antibiotics at intermediate-to-long periods. **(P-T) High constitutive Bla production. (P) Time course**. Here, bacteria constitutively produce Bla at a rate an order of magnitude greater than the bacteria that constitutively produce low amounts of Bla. As a result, the bacteria produce sufficient Bla such that the private Bla present in the periplasm can provide sufficient protection against the antibiotic. As such, the overall population growth rate is always greater than the lysis rate; thus the population will always increase in density. **(Q) Recovery time**. Because the bacteria produce such high amounts of Bla, the population experiences no or little initial decrease in density. Thus, the recovery time is close to 0 until stronger antibiotic concentrations are applied. **(R) Final density**. Protocols using periods of less than one recovery time are effective at clearing an infection, as long as the antibiotic concentration induces a sufficiently long recovery time (see S5 Fig). **(S) Dose number**. The shorter periods are associated with increased number of doses because there would be less time between when cells lyse and the Bla will not have the chance to naturally degrade before the next round of lysis adds more Bla to the environment. Higher concentrations and longer period lengths result in lower dose numbers. **(T) Total antibiotic delivered**. The least amount of antibiotic delivered is associated with regimens using lower dose concentrations and intermediate-to-long periods. This supports the notion that longer periods allow for the large amounts of Bla to degrade before the next dose, thus allowing each dose to be as effective as possible. **(U-Y) High constitutive Bla production, IV-drip treatment. (U) Time course**. The antibiotic was infused into the system over 3 time units, resulting in a sustained high concentration that led to a sustained decline in the population density. The Bla produced was insufficient to effectively degrade the antibiotic while it was being infused, so the population could not start to recover until after the infusion had ended. **(V) Recovery time**. A higher concentration of antibiotic was necessary to elicit a recovery time. Relative to the population constitutively producing Bla that was treated with an injection regimen, the IV-drip treatment produced longer recovery times. **(W) Final density**. IV-drip therapy results support the predictive power of the recovery time for populations constitutively producing high levels of Bla. Doses delivered at periods less than 1 recovery time clear the infection whereas doses delivered at periods longer than 1 recovery time fail. **(X) Dose number**. The fewest doses are required for regimens using high antibiotic concentrations delivered at intermediate-long periods. **(Y) Total antibiotic delivered**. The least amount of antibiotic was delivered for regimens delivering the intermediate concentrations of antibiotic at intermediate-to-long periods.
(PDF)

S4 Fig Predictive power is lost when single dose recovery time does not predict recovery time of subsequent doses. **(A-B) Population recovers at periods < 1 recovery time. (A) Final Density**. Populations can recover from an antibiotic applied at periods less than one recovery time if the bacteria are producing extreme levels of Bla and the antibiotic concentration is too weak to induce sufficient lysis (i.e. has a short recovery time). If the recovery time is too short, then the time between doses is too short for the Bla to return to a baseline level. As a result, the net Bla compounds with each subsequent dose, allowing populations to survive an antibiotic applied at periods less than one recovery time. **(B)**. **Time curves for cell density, Bla, and antibiotic concentration**. Because each subsequent dose of antibiotic causes more cells to lyse and release Bla before the Bla from the previous dose is degraded, there is an increase in the base amount of Bla always present. As a result, the cells can clear the antibiotic faster on subsequent doses compared to the first dose. Consequentially, the observed recovery time from the multi-dose regimen is actually shorter than the expected response time calculated from the single dose. Here, A = 1.4 and period = 0.60 RT. **(C-D) Population fails to recover at periods = 1 recovery time. (C) Final Density**. Populations fail to recover from a low antibiotic concentration applied at periods less than one recovery time if the bacteria are producing low levels of Bla. Because the antibiotic concentration is low, the recovery time and its derived dosing periods are short. The time between doses is insufficient for the Bla to degrade enough of the antibiotic to recover. As a result, the antibiotic concentration compounds with each subsequent dose, preventing populations from recovering at periods greater than one recovery time. **(D)**. **Time curves for cell density, Bla, and antibiotic concentration**. With each subsequent dose of antibiotic, more cells lyse and release Bla; however, the cells produce insufficient Bla to degrade the current dose of antibiotic before the next dose is delivered. As a result, the antibiotic accumulates with each subsequent dose and the cells are killed. Here, A = 0.63 and period = 1.33 RT.
(PDF)

S5 Fig The effect of persisters on the predictive powers of recovery time. Our model assumes that persisters form a small fraction of a population, grow and lyse at rates much slower than normal cells (*g_p_* ≪ *g_N_*, *l_P_* ≪ *l_N_*), and, when the antibiotic concentration is low enough (*a* < *σ_1_*), they are generated from and revert to a normal cell phenotype at the slow rates of *κ_P_* and *κ_N_*, respectively. **Population with persisters that grow and lyse at rates 100 times more slowly than normal cells (A-E). (A) Time course**. The bacteria lyse due to the antibiotic, release Bla to degrade the antibiotic, and then recover once the antibiotic concentration is low enough. At this point, persisters (dashed black line) are generated from and return to the normal cell population (solid black line). Here, the initial density of persisters and normal cells are 0.00001 and 0.1, respectively. **(B) Recovery time**. The recovery times are the same between the population containing persisters (black line) and the population containing no persisters (grey line) until. *a*_0_ > 0.3. From 0.3 < *a*_0_ < 26, both populations take longer to recover with increasing antibiotic concentration; however, the population containing persisters recovers slightly faster. When *a*_0_ > 26, then the population without persisters fails to recover, whereas the population with persisters is able to re-establish the population. **(C) Final Density**. After 100 doses of antibiotic, the final total density was measured. Periods greater than 1 recovery time resulted in the full recovery of the population; however, periods less than 1 recovery time appeared to suppress the population’s recovery. **(D) Dose number**. For each antibiotic concentration and period combination, the corresponding number of doses necessary to reduce the total population density to a critical threshold was calculated. The regimens using antibiotics associated with longer recovery times and longer periods require the fewest doses. **(E) Total antibiotic delivered**. Despite many different regimens requiring the same number of doses, the regimens could be differentiated by the total amount of antibiotic delivered. When comparing regimens with the same number of doses, the amount of antibiotics delivered would decrease as the dose concentration decreased. **Population with persisters that grow and lyse at rates 1000 times more slowly than normal cells (F-J). (F) Time course**. Similar to **(A)**. **(G) Recovery time**. Similar to **(B)**. **(H) Final Density**. Similar to general trend of **(C)**, where final densities are high when using periods > 1 recovery time and are low otherwise. However, many of the low final densities achieved after 100 doses with periods < 1 recovery time are close to, but not exactly, 0. This means that once the regimen is completed and sufficient time has passed, the population will be able to regrow. **(I) Dose number**. For each antibiotic concentration and period combination, the corresponding number of doses necessary to reduce the total population density to a critical threshold was calculated. The regimens using antibiotics associated with longer recovery times and longest periods require the fewest doses. The regimens using lower concentrations of antibiotic (*a*_0_ < 10) and/or shorter dosing periods (period < 0.65 recovery time) were not successful at reducing the population below the threshold after 100 doses. This is due to the presence of persisters. **(J) Total antibiotic delivered**. The total amount of antibiotic delivered for each regimen was calculated and suggests that an injection based regimen would need very high concentrations of antibiotic to sufficiently reduce a population with persisters. **Schematic for how a population with persisters responds to antibiotic**. Black represents bacterial actions, blue represents Bla actions, and red represents antibiotic actions. Arrows denote induction or activation; T-lines indicate inhibition; the dashed arrow represents the ability for the model to simulate inducible or constitutive Bla production.
(PDF)

S6 Fig The effect of a mixed population on the predictive powers of recovery time. The model represents a mixed population with equal starting densities of two subpopulations (*n*_1_ and *n*_2_) with different levels of antibiotic resistance. *n*_1_ has the same parameter values as the base case from the homogeneous model; however, *n*_2_ has increased thresholds for antibiotic effects. Particularly, *n*_2_ requires higher concentrations of antibiotic to inhibit growth (*σ*_5_ = 5*σ*_1_) and induce lysis (*σ*_6_ = 5*σ*_2_). Although this model accounts for two distinct subpopulations, it could be extended to multiple populations displaying some degree of collective antibiotic tolerance. **Subpopulation can switch between states (A-E). (A) Time course**. Both subpopulations start at the same starting density (0.05) and start to lyse due to antibiotic. One subpopulation (*n*_2_) is more resistant than the other (*n*_1_), with thresholds for growth inhibition (*σ*_5_) and lysis (*σ*_6_) 5 times higher. Both populations contribute Bla to degrade the antibiotic. Once the antibiotic concentration is sufficiently low, then the subpopulations can start to recover. Since *n*_1_ and *n*_2_ can switch between states, *n*_2_ can help *n*_1_ recover under concentrations that would otherwise be lethal. **(B) Recovery time curve**. Because both populations still undergo the process of lysing before recovering, the dosing protocol based on recovery time is still applicable. When 0.3 < *a*_0_ < 2.3, both *n*_1_ and *n*_2_ are recovering at similar rates, thus the population as a whole recovers quickly. Once 2.3 < *a*_0_ < 26, then *n*_2_ recovers faster and determines the population level recovery time. When *a*_0_ < 26, then both subpopulations cannot recover. **(C) Final density**. Protocols using periods of less than one recovery time are effective at eliminating the population, regardless of antibiotic concentration. **(D) Dose number**. For each antibiotic concentration and period combination, the corresponding number of doses necessary to eliminate the population was calculated. The regimens using antibiotics associated with longer recovery times require the fewest doses; however, lower concentrations of antibiotic (10 < *a*_0_ < 26)can be effective if applied at period lengths of 0.10–0.50 period/RT. **(E) Total antibiotic delivered**. Despite many different regimens requiring the same number of doses to clear an infection, the regimens could be differentiated by the total amount of antibiotic delivered. When comparing regimens with the same number of doses, the amount of antibiotics delivered would decrease as the dose concentration decreased. **Subpopulations cannot switch between states (F-J). (F) Time course**. Same as in **(A)** except *n*1 and *n*_2_ do not switch back and forth. Thus the more resistant subpopulation, *n*_2_, is selected to recover. **(G) Recovery time curve**. Same as in **(B)**. **(H) Final density**. Same as in **(C)**. **(D) Dose number**. Same as in **(D). (J) Total antibiotic delivered**. Same as in **(E)**. **Schematic for a mixed population’s response to antibiotic**. Black represents bacterial actions, blue represents Bla actions, and red represents antibiotic actions. Arrows denote induction or activation; T-lines indicate inhibition; the dashed arrow represents the ability for the model to simulate inducible or constitutive Bla production.
(PDF)
