# Supplementary material for: Bacterial Temporal Dynamics Enable Optimal Design of Antibiotic Treatment
Source: PLoS Comput Biol. 2015 Apr 23;11(4):e1004201. doi: 10.1371/journal.pcbi.1004201 (PMC4407907; doi:10.1371/journal.pcbi.1004201)
Supplement: S1 Text — The equations, nondimensionalizing terms, and parameter values are detailed here for the different models. (DOCX) [file pcbi.1004201.s001.docx]

**S1 Text: Model Development**

The interaction between a β-lactam and a bacterial population expressing a β-lactamase (Bla) can be simplified to the interactions between three main components: population density, antibiotic concentration, and Bla concentration. To model bacteria that inducibly or constitutively produce Bla and lyse due to antibiotics degrading the cell wall, we used an ordinary-differential-equation model for the dynamics of bacterial density (*N*), Bla concentration (*B*), and β-lactam concentration (*A*).

**Dimensional EQs:**

$$\frac{dN}{dt}=\left( G-L \right)N$$

$$\frac{dB_{out}}{dt}=LB_{in}^{*}-d{}_{B_{out}}B_{out}-k_{IV}\left( t \right)B_{out}$$

$$\frac{dA}{dt}=k_{IV}(t)A_{inject}-k_{B_{out}}\left( B_{out}+\alpha B_{in}^{*} \right)\left( \frac{A}{K{}_{A}+A} \right)-d_{A}A-k_{IV}(t)A$$

$$G=\left( 1-\frac{N}{N_{m}} \right)\left( \frac{\mu K_{1}}{K_{1}+A} \right)$$

$$L=\left( \frac{d{}_{N}A^{H}}{K_{2}^{H}+A^{H}} \right)\left( \frac{K_{4}}{K_{4}+B_{in}} \right)$$

$B_{in}=\frac{k{}_{B_{in}}R}{G+d_{B_{in}}}$

$$B_{in}^{*}=B_{in}V_{cell}N$$

$$R=\frac{A}{K_{3}+A}$$

**Our key modeling assumptions are:**

- The dynamics of cell density (*N*) depend on the growth rate (*G*) and the lysis rate (*L*).
- *G* is a function of *N*, the bacterial carrying capacity (*N_m_*), the specific growth rate of the cells (*µ*), the half-maximal constant for growth inhibition by the antibiotic (*K_1_*), and the antibiotic concentration (*A*).
- *L* is a function of the maximum lysis rate constant (*d_N_*), the antibiotic concentration (*A*), the half maximal constant cell lysis by the antibiotic (*K_2_*), the Hill coefficient (*H*), the half-maximal constant for antibiotic degradation by the periplasmic Bla (*K_4_*), and the concentration of periplasmic Bla (*B_in_*).
- The dynamics of extracellular Bla present in the culture (*B­_out_*) depends on how much Bla is released, degraded (with a rate constant, *d_Bout_*), and washed out of the system (with an IV flow rate, *k_IV_* (e.g. $\frac{1}{pulse length}$ ), when applicable). Bla release depends on *L*, the concentration of intracellular Bla (*B_in_*), *N*, and the volume of a cell (*V_cell_*).
- The dynamics of the intracellular Bla (*B_in_*) is a function of the maximum production rate of Bla per cell (*k­_Bin_*), the rate of Bla induction (*P*), *G*, and the intracellular degradation rate constant of Bla (*d­_Bin_*). *P* is a function of *A* and the half maximal constant for antibiotics inducing the production of Bla (*K_3_*). If *K_3_* is set to 0, Bla production is constitutive; if *K_3_* is greater than 0, the Bla production is inducible by *A*.
- The different methods of antibiotic delivery (injection versus IV drip) were differentiated by the time dependent flow rate, *k_IV_(t)*. It would be 0 for injections (delivered instantaneously) and non-zero for IV drips (delivered over a set time). It was assumed that the rate of antibiotic being introduced by IV would be the same rate at which the antibiotic would be pushed out of the region. The IV drip duration was determined by a step function dependent on time.
- The dynamics of antibiotic concentration in the environment is dependent on the amount of Bla present (both *B­_out_* and *B_in_*), how quickly Bla degrades the β-lactam (*k_Bout_*), A, the half-maximal constant for degradation of the antibiotic by Bla (*K_A_*), and the degradation rate constant of the antibiotic (*d­_A_*).
- To convert periplasmic Bla from a per cell basis (*B_in_*) to a per population basis (*B_in_^*^*), *B_in_* was multiplied by the number of cells present at a given time and the volume of a cell. The efficacy of periplasmic Bla is altered by a weighting variable (*α*).
- In these models, the antibiotic has two main effects: inhibiting cell growth and promoting lysis (Figure 1A). In the inducible model, antibiotics also serve to activate the production of a Bla.

Non-dimensionalizing the equations by $n=\frac{N}{N_{m}}, b_{out}=\frac{B_{out}k_{Bout}}{\mu K_{A}}, a=\frac{A}{K_{A}}, and \tau=t\mu$ gives

$\frac{dn}{d\tau}=\left( g-l \right)n$

$\frac{db_{out}}{d\tau}=lb_{in}^{*}-\gamma_{2}b_{out}-\kappa_{IV}\left( \tau\right)b_{out}$

$\frac{da}{d\tau}=\kappa_{IV}{\left( \tau\right)a}_{inject}-\left( b_{out}+\alpha b_{in}^{*} \right)\left( \frac{a}{1+a} \right)-\gamma_{3}a-\kappa_{IV}(\tau)a$

$g=\left( 1-n \right)\left( \frac{\sigma_{1}}{\sigma_{1}+a} \right)$

$l=\gamma_{1}\left( \frac{a^{H}}{\sigma_{2}^{H}+a^{H}} \right)\left( \frac{\sigma_{4}}{\sigma_{4}+b_{in}} \right)$

$b_{in}=\kappa\left( \frac{r}{g+\gamma_{4}} \right)$

$b_{in}^{*}=\beta{nb}_{in}$

$r=\frac{a}{\sigma_{3}+a}$

where $\sigma_{1}=\frac{K_{1}}{K_{A}}, \sigma_{2}=\frac{K_{2}}{K_{A}}, \sigma_{3}=\frac{K_{3}}{K_{A}},{\sigma_{4}=\left( \frac{K_{4}k_{B_{out}}}{K_{A}\mu} \right),\gamma}_{1}=\frac{d_{N}}{\mu}, \gamma_{2}=\frac{d_{B_{out}}}{\mu}, \gamma_{3}=\frac{d_{A}}{\mu},\gamma_{4}=\frac{d_{B_{in}}}{\mu}, \beta=N_{m}V_{cell}, \kappa_{IV}=\frac{k_{IV}}{\mu}, and \kappa=(\frac{k_{b_{in}}k_{b_{out}}}{\mu^{2}K\_A})$.

**Heterogeneous population model:**

To model a heterogeneous population, we modified the model described above to account for a second population. By adjusting the maximum lysis ($\gamma_{5}$) and growth ($\nu$) rates of the second subpopulation, this model can account for the dynamics of persisters (*P*), a small fraction of persisters that grow and lyse significanlty more slowly than normal cells and revert back to/generate from normal cells once the antibiotic concentration is sufficiently low [[1](#_ENREF_1),[2](#_ENREF_2)]. This same model can also be used to represent a population consisting of bacteria with different levels of antibiotic resistance by adjusting the thresholds for growth inhibition ($\sigma_{5}$) and lysis ($\sigma_{6}$). This model could ultimately be expanded to account for multiple subpopulations, given that they all displayed some level of collective antibiotic tolerance:

$$\frac{dN}{dt}=\left( G-L \right)N+k_{N}P-k_{p}N$$

$$\frac{dP}{dt}=\left( G_{P}-L_{P} \right)P +k_{P}N-k_{N}P$$

$$\frac{dB_{out}}{dt}=(L+L_{P})B_{in}^{*}-d{}_{B_{out}}B_{out}-k_{IV}\left( t \right)B_{out}$$

$$\frac{dA}{dt}=k_{IV}(t)A_{inject}-k_{B_{out}}\left( B_{out}+\alpha B_{in}^{*} \right)\left( \frac{A}{K{}_{A}+A} \right)-d_{A}A-k_{IV}(t)A$$

$$G=\left( 1-\frac{N{}_{T}}{N_{m}} \right)\left( \frac{\mu K_{1}}{K_{1}+A} \right)$$

$$G_{P}=\left( 1-\frac{N_{T}}{N_{m}} \right)\left( \frac{\mu_{p}K_{5}}{K_{5}+A} \right)$$

$$L=\left( \frac{d{}_{N}A^{H}}{K_{2}^{H}+A^{H}} \right)\left( \frac{K_{4}}{K_{4}+B_{in}} \right)$$

$$L_{P}=\left( \frac{d{}_{P}A^{H}}{K_{6}^{H}+A^{H}} \right)\left( \frac{K_{4}}{K_{4}+B_{in}} \right)$$

$B_{in}=\frac{k{}_{B_{in}}R}{G+G_{p}+d_{B_{in}}}$

$B_{in}^{*}=B_{in}V_{cell}N_{T}$

$$R=\frac{A}{K_{3}+A}$$

$$N{}_{T}=N+P$$

**Our key modeling assumptions for the persister model are:**

- The dynamics of cell density (*N*) depends on the growth rate (*G*), the lysis rate (*L*), the rate at which persisters (*P*) are generated from the normal cell population ($k_{P})$, and the rate at which persisters return to the normal cell phenotype ($k_{N})$. We assume that $k_{N}=k_{P}=0$ until *A* is sufficiently low enough ($a<\sigma_{1}$), after which $k_{N}\gg k_{P}$.
- The dynamics of persister cell density (*P*) depend on the persisters’ growth rate ($G_{P}$), lysis rate ($L_{P}$), rate of generation ($k_{P}$) from normal cells ($N$), and rate of reversion back to *N* ($k_{N}$).
- The dynamics of extracellular Bla present in the culture (*B­_out_*) is now a function of both the normal cell lysis rate (*L*) and the persister cell lysis rate (*L­_P_*), the concentration of intracellular Bla (*B_in_*), the total number of cells (*N­­_T_*), and the volume of a cell (*V_cell_*).
- $G_{P}$*­* is a function of $N_{T}, N_{M}$*,* the specific growth rate of the cells ($\mu_{P}$), the half-maximal constant for growth inhibition by the antibiotic (*K_5_*), and the antibiotic concentration (*A*). For persisters, we assume that the threshold for growth inhibition is the same as the normal cell’s ($K_{1}=K_{5})$ and that the growth rate is the distinguishing factor.
- $L_{P}$*­* is a function of the maximum lysis rate constant of persisters ($d_{p}$), the antibiotic concentration (*A*), the half maximal constant cell lysis by the antibiotic (*K_6_*), the Hill coefficient (*H*), the half-maximal constant for antibiotic degradation by the periplasmic Bla (*K_4_*), and the concentration of periplasmic Bla (*B_in_*). For persisters, we assume that the threshold for lysis is the same as the normal cell’s ($K_{2}=K_{6})$ and that the lysis rate is the distinguishing factor.
- $B_{in}$and $B_{in}^{*}$ have been updated to reflect both populations’ contributions.

**Our key modeling assumptions for a mixed population model are:**

- To model a mixed population with distinct subpopulations, set $k_{P}=k_{N}=0$.
- We assume that the distinguishing features between populations are the thresholds for growth inhibition ($\sigma_{1},\sigma_{5}$) and lysis ($\sigma_{2},\sigma_{6}$). By altering the relative values of these parameters ($\sigma_{1}<\sigma_{5}, \sigma_{2}<\sigma_{6}$), the dynamics of populations with different resistance levels can be modeled.

Non-dimensionalizing the equations by $P=\frac{P}{N_{m}}$ gives

$\frac{dn}{d\tau} =(g-l)n+\kappa_{N}p-\kappa_{P}n$

$\frac{dp}{d\tau}={(g}_{P}-l{}_{p})p+\kappa_{p}n -\kappa_{N}p$

$\frac{db_{out}}{d\tau}=(l+l_{P})b_{in}^{*}-\gamma_{2}b_{out}-\kappa_{IV}\left( \tau\right)b_{out}$

$\frac{da}{d\tau}=\kappa_{IV}{\left( \tau\right)a}_{inject}-\left( b_{out}+\alpha b_{in}^{*} \right)\left( \frac{a}{1+a} \right)-\gamma_{3}a-\kappa_{IV}(\tau)a$

$g=\left( 1-n_{T} \right)\left( \frac{\sigma_{1}}{\sigma_{1}+a} \right)$

$g_{p}=\left( 1-n_{T} \right)\left( \frac{\sigma_{5}}{\sigma_{5}+a} \right)\upsilon$

$l=\gamma_{1}\left( \frac{a^{H}}{\sigma_{2}^{H}+a^{H}} \right)\left( \frac{\sigma_{4}}{\sigma_{4}+b_{in}} \right)$

$l_{p}=\gamma_{5}\left( \frac{a^{H}}{\sigma_{6}^{H}+a^{H}} \right)\left( \frac{\sigma_{4}}{\sigma_{4}+b_{in}} \right)$

$b_{in}=\kappa\left( \frac{r}{g+g_{p}+\gamma_{4}} \right)$

$b_{in}^{*}=\beta b_{in}n_{T}$

$r=\frac{a}{\sigma_{3}+a}$

$$n_{T}=n+p$$

where $\sigma_{5}=\frac{K_{5}}{K_{A}}, \sigma_{6}=\frac{K_{6}}{K_{A}},\gamma_{5}=\frac{d_{P}}{\mu}, \kappa_{N}=\frac{k_{N}}{\mu},\kappa_{P}=\frac{k_{P}}{\mu}, and \upsilon=\frac{\mu_{p}}{\mu}$.

| Parameter | Description and Source | Value |
| --- | --- | --- |
| $\alpha$ | Weighting factor for periplasmic β-lactamase, which provides less protection against antibiotic than extracellular β-lactamase. | 0.01 |
| $H$ | Hill coefficient for determining lysis rate. A low Hill coefficient ($H$=1) represents an antibiotic with a dose-dependent lysis rate, whereas a higher Hill coefficient ($H$=3) represents an antibiotic with a time-dependent lysis rate. [[3](#_ENREF_3)] | 3 (1) |
| $\beta=\left( N_{m}V_{cell} \right)$ | Parameter used to convert from single cell level to population level where $N_{m}$ = 10^9^ cells/mL and $V_{cells}$= 10^-12^ mL/cell. [[4](#_ENREF_4)] | 0.001 |
| $\gamma_{1}=\left( \frac{d_{N}}{\mu} \right)$ | Maximum lysis rate by antibiotic given that $\mu$=0.8/hour.[[5](#_ENREF_5)] Tuomanen et al. suggest that 50-90% of a population can lyse per hour ($d_{N}$).[[6](#_ENREF_6)] | 62 |
| $\gamma_{2}$*=*$\left( \frac{d_{B_{out}}}{\mu} \right)$ | Maximum degradation rate of extracellular Bla given that $d_{B_{out}}$=0.46/hour. [[7](#_ENREF_7)] | 0.58 |
| $\gamma_{3}=\left( \frac{d_{A}}{\mu} \right)$ | Maximum degradation rate of β-lactam antibiotic, given that $d_{A}$=0.35/hour. [[8](#_ENREF_8),[9](#_ENREF_9)] | 0.51 |
| $\gamma_{4}=\left( \frac{d_{b_{in}}}{\mu} \right)$ | Maximum degradation rate of periplasmic Bla given that $d_{B_{in}}$=0.2/hour. [[10](#_ENREF_10)] | 0.25 |
| $\gamma_{5}=\left( \frac{d_{P}}{\mu} \right)$ | Maximum lysis rate of the second population by antibiotic given that $\mu$=0.8/hour. The persister model assumes that $d_{P}$ is 2-3 orders of magnitude smaller than $d_{N}$. The mixed population model assumes that $d_{P}=d_{N}$. | 0.062:62 |
| $\nu=\left( \frac{\mu_{P}}{\mu} \right)$ | Maximum growth rate of the second population, given that *μ*=0.8/hour. The persister model assumes that $\mu_{P}$ is 2-3 orders of magnitude smaller than $\mu$ for the situations when persisters are practically not growing and 0.02 for the situations when persisters are slowly growing[[1](#_ENREF_1),[2](#_ENREF_2)]. The mixed population model assumes that $\mu_{P}=\mu$. | 0.001:1 |
| $\kappa_{IV}=\left( \frac{k_{IV}}{\mu} \right)$ | Rate of IV drip given that the dose is delivered over a set pulse length (here, pulse length was arbitrarily set to 2.4 hours). | 0.33 |
| $\kappa=\left( \frac{k_{b_{in}}k_{B_{out}}}{\mu^{2}K_{A}} \right)$ | Efficiency of Bla is determined by how quickly Bla is produced and how efficiently Bla can hydrolyze a β-lactam antibiotic. The maximum rate Bla can be produced (*k­_Bin_*) is on the order of 0.1 μM/hour [[11](#_ENREF_11)], the maximum rate of (*k_Bout_*) is on the order of 1E10^5^-1E10^6^/hour (variable, depending on which antibiotic used) [[11](#_ENREF_11),[12](#_ENREF_12)]. | 5.44E3 |
| $\sigma_{1}=\left( \frac{K_{1}}{K_{A}} \right)$ | Half maximal constant for growth inhibition, given that *K_1_*= 0.2-16 μM [[13](#_ENREF_13)]and *K_A_*=33-150 μM [[12](#_ENREF_12)] (depending on the antibiotic). | 0.24 |
| $\sigma_{2}=\left( \frac{K_{2}}{K_{A}} \right)$ | Half maximal constant for lysis by antibiotic, assuming that *K_2_* is larger than *K_1_* (there is a small range of concentrations under which cells have stopped growing, but are not lysing). | 1.29 |
| $\sigma_{3}=\left( \frac{K_{3}}{K_{A}} \right)$ | Half maximal constant for inducing Bla production, assuming that it takes much higher concentrations for bacteria to produce Bla than it would for bacteria to stop growing or start lysing. [[14](#_ENREF_14)] | 5.17 or 0 |
| $\sigma_{4}=\left( \frac{K_{4}k_{B_{out}}}{K_{A}\mu} \right)$ | Half maximal constant for periplasmic Bla protection, assuming that very little periplasmic Bla is necessary for a single cell to experience a small amount of protection from the antibiotic. | 2.08E3 |
| $\sigma_{5}=\left( \frac{K_{5}}{K_{A}} \right)$ | Half maximal constant for growth inhibition of the second population. Persister cells are assumed to have a similar threshold as normal cells ($K_{5}=K_{1}$), whereas as more resistant population would have a higher threshold ($K_{5}={5K}_{1}$). | 0.24:1.2 |
| $\sigma_{6}=\left( \frac{K_{6}}{K_{A}} \right)$ | Half maximal constant for lysis by antibiotic of the second population Persister cells are assumed to have a similar threshold as normal cells ($K_{6}=K_{2}$), whereas as more resistant population would have a higher threshold ($K_{6}={5K}_{2}$). | 1.29:6.45 |

**References:**

1. Balaban NQ, Merrin J, Chait R, Kowalik L, Leibler S (2004) Bacterial persistence as a phenotypic switch. Science 305: 1622-1625.

2. Johnson PJ, Levin BR (2013) Pharmacodynamics, population dynamics, and the evolution of persistence in Staphylococcus aureus. PLoS genetics 9: e1003123.

3. Czock D, Keller F (2007) Mechanism-based pharmacokinetic–pharmacodynamic modeling of antimicrobial drug effects. Journal of pharmacokinetics and pharmacodynamics 34: 727-751.

4. Milo R (2010) BioNumbers: Useful Fundamental Numbers in Molecular Biology. Nucleic Acids Research 38: 3.

5. Tanouchi Y, Pai A, Buchler NE, You L (2012) Programming stress-induced altruistic death in engineered bacteria. Molecular systems biology 8.

6. Tuomanen E, Cozens R, Tosch W, Zak O, Tomasz A (1986) The Rate of Killing of Escherichia coli byβ-Lactam Antibiotics Is Strictly Proportional to the Rate of Bacterial Growth. Journal of general microbiology 132: 1297-1304.

7. Wu X-C, Lee W, Tran L, Wong S (1991) Engineering a Bacillus subtilis expression-secretion system with a strain deficient in six extracellular proteases. Journal of bacteriology 173: 4952-4958.

8. Bergan T (1983) Pharmacokinetics of beta-lactam antibiotics. Scandinavian journal of infectious diseases Supplementum 42: 83-98.

9. Drusano GL (1988) Role of pharmacokinetics in the outcome of infections. Antimicrobial Agents and Chemotherapy 32: 289.

10. Zlokarnik G, Negulescu PA, Knapp TE, Mere L, Burres N, et al. (1998) Quantitation of transcription and clonal selection of single living cells with β-lactamase as reporter. Science 279: 84-88.

11. Vu H, Nikaido H (1985) Role of beta-lactam hydrolysis in the mechanism of resistance of a beta-lactamase-constitutive Enterobacter cloacae strain to expanded-spectrum beta-lactams. Antimicrobial agents and chemotherapy 27: 393-398.

12. Osuna J, Viadiu H, Fink AL, Soberón X (1995) Substitution of Asp for Asn at Position 132 in the Active Site of TEM-Lactamase ACTIVITY TOWARD DIFFERENT SUBSTRATES AND EFFECTS OF NEIGHBORING RESIDUES. Journal of Biological Chemistry 270: 775-780.

13. O'Callaghan CH, Morris A (1972) Inhibition of β-Lactamases by β-Lactam Antibiotics. Antimicrobial agents and chemotherapy 2: 442-448.

14. Giwercman B, Jensen E, Høiby N, Kharazmi A, Costerton J (1991) Induction of beta-lactamase production in Pseudomonas aeruginosa biofilm. Antimicrobial agents and chemotherapy 35: 1008-1010.
