# Supplementary figures and images for: Bacterial Temporal Dynamics Enable Optimal Design of Antibiotic Treatment
Source: PLoS Comput Biol. 2015 Apr 23;11(4):e1004201. doi: 10.1371/journal.pcbi.1004201 (PMC4407907; doi:10.1371/journal.pcbi.1004201)

$N_0=0.01$

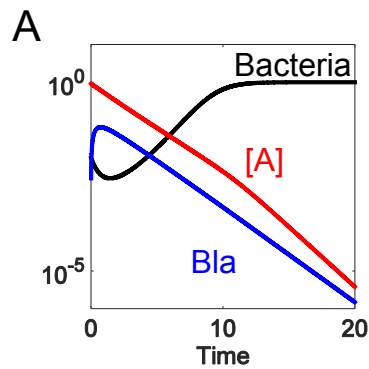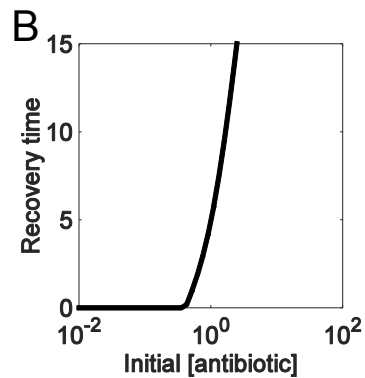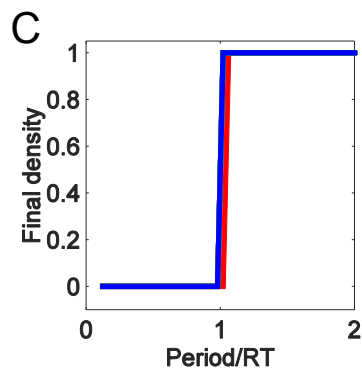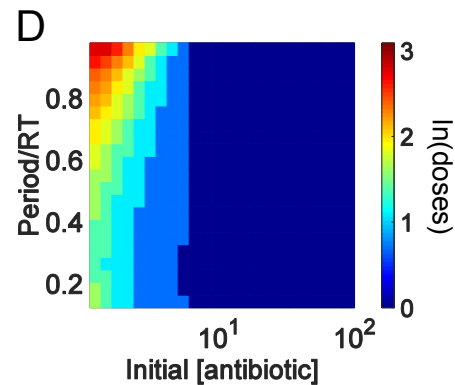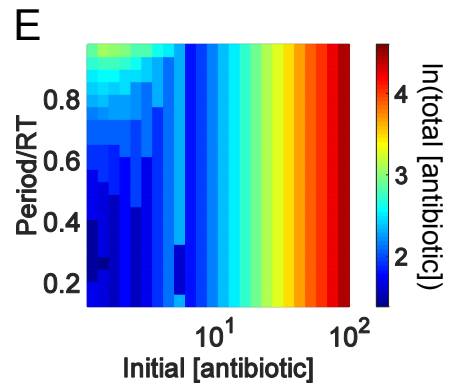

$N_0=0.001$

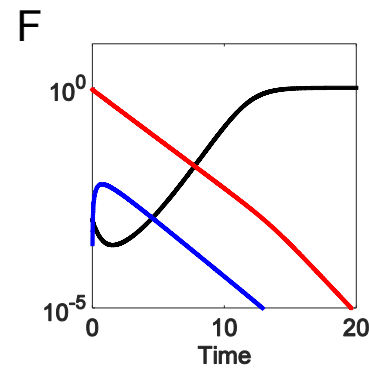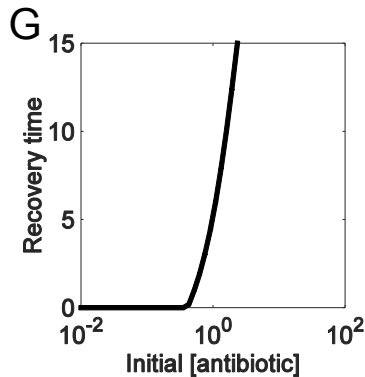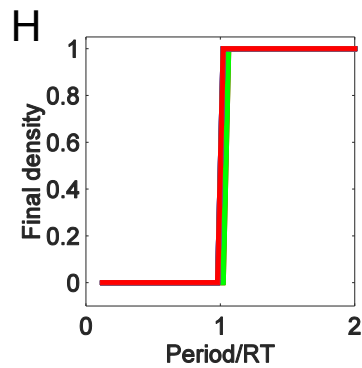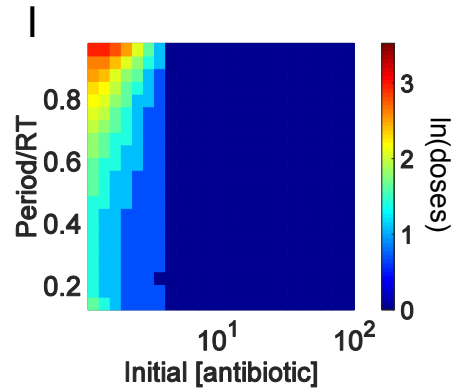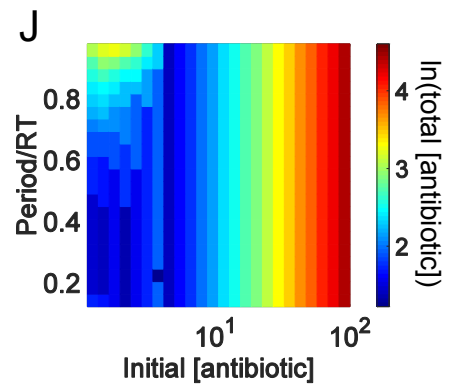

Supplement: S1 Fig — (A,F) Time courses for populations with initial densities that are 10x and 100x smaller than the base model. (B,G) Recovery time curves for populations with initial densities that are 10x and 100x smaller than the base model. (C,H) Final density depends on dosing frequency. Despite the lower initial densities, both models followed the trend where periods less than one recovery time eliminate the population as long as the initial antibiotic concentration is sufficiently high to cause significant initial decline. This indicates that the recovery time is a viable tool for predicting treatment outcomes for a range of population sizes. (D,I) Dose number necessary to reduce a population below critical threshold depends on antibiotic concentration and period length. The fewest number of doses corresponds to the antibiotic concentrations with the longest recovery times; however, intermediate concentrations can be effective when applied at low to intermediate period lengths. (E,J) Total antibiotic concentration delivered depends on single dose concentration. The regimens applying doses of lower concentrations of effective antibiotic will eliminate the population just as effectively as the regimens using high concentrations, but with less total antibiotic. (PDF) [file pcbi.1004201.s002.pdf]

Hill=1

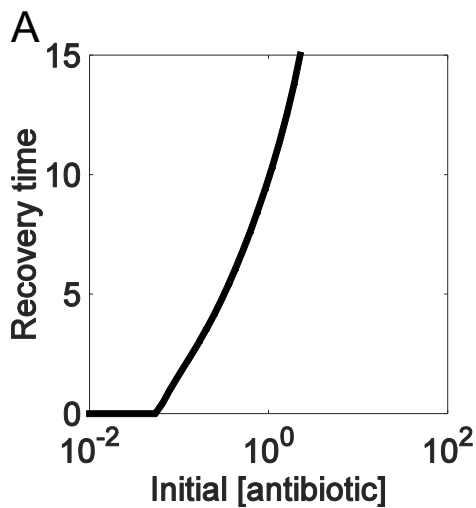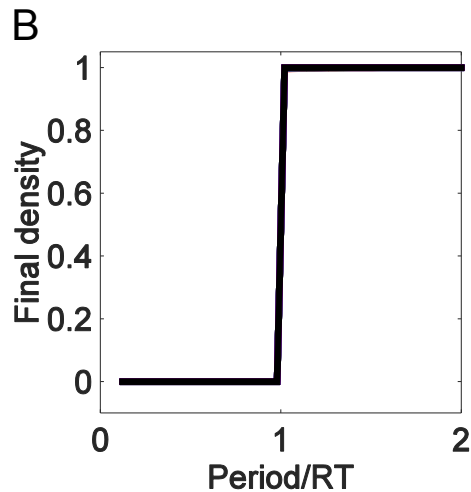

Hill=10

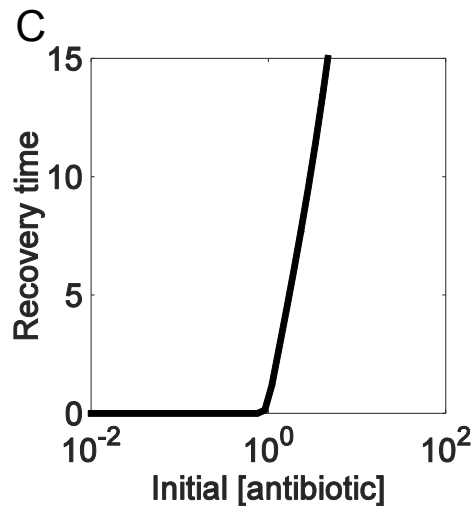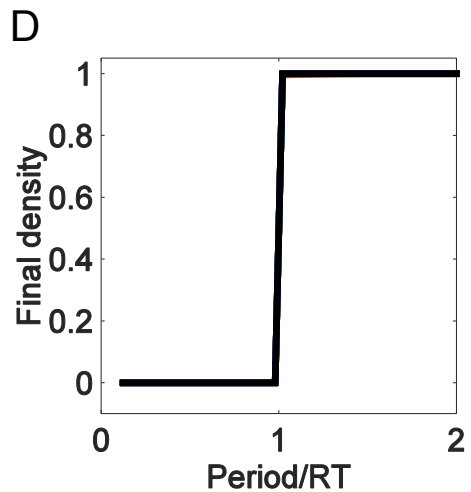

Supplement: S2 Fig — (A,C) Recovery time depends less on antibiotic concentration if the Hill coefficient (H) is high enough. When H = 1, the recovery time is dose dependent, increasing as the antibiotic concentration increases. When H = 10, the recovery time quickly transitions from being 0 to infinite (the population has been wiped out). Once past the threshold, increasing the antibiotic concentration will not continue to increase the recovery time. (B,D) Final density depends on dosing frequency. Despite the different Hill coefficients, both models followed the trend where periods less than one recovery time eliminate the population as long as the initial antibiotic concentration is sufficiently high to cause significant initial decline. This indicates that the recovery time is a viable tool for predicting treatment outcomes for a range of antibiotics with different modes of killing (time vs. dose dependent). (PDF) [file pcbi.1004201.s003.pdf]

No Bla

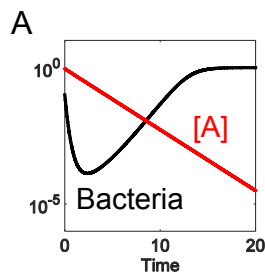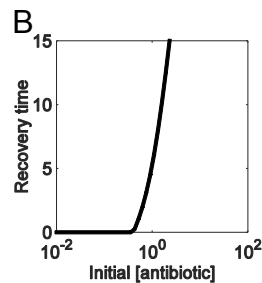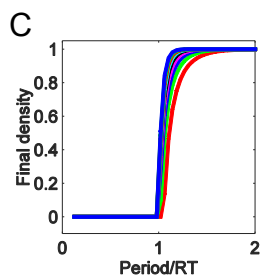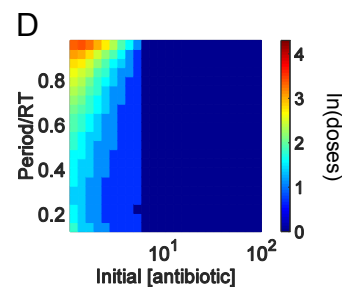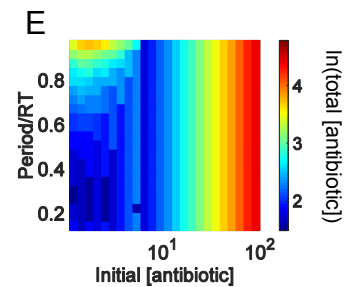

Low Inducible

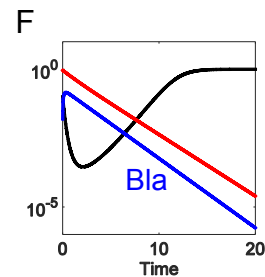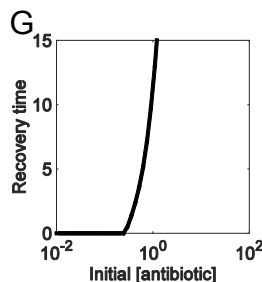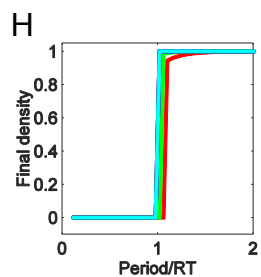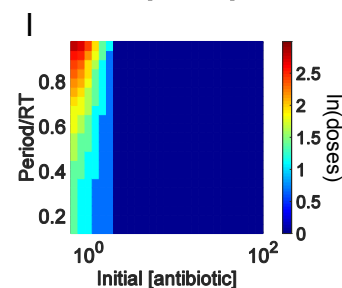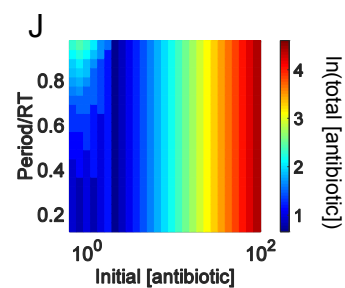

High Inducible

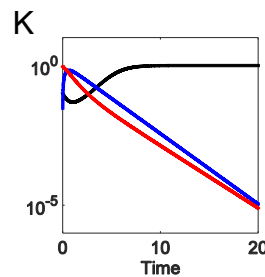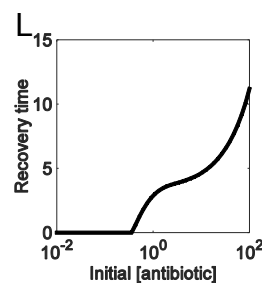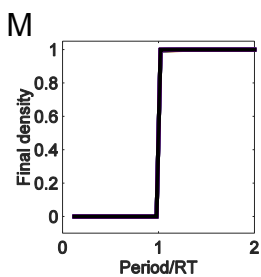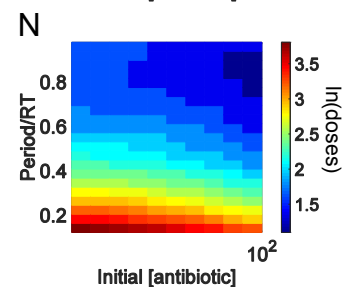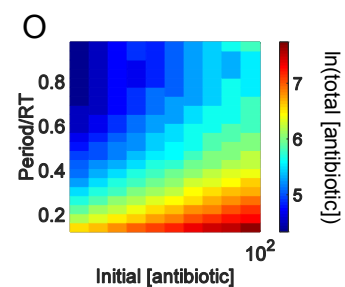

High Constitutive

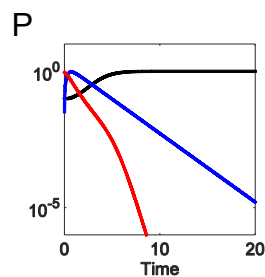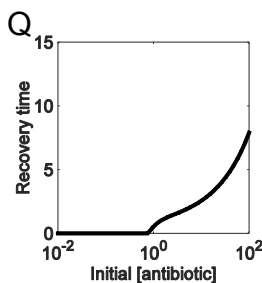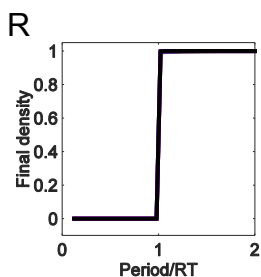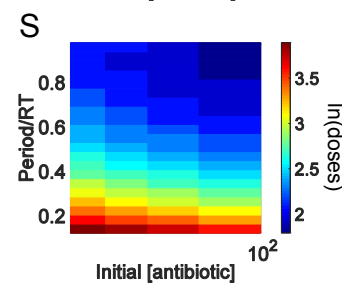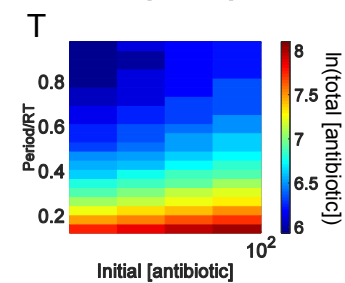

High Constitutive,  
IV drip

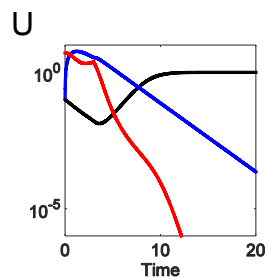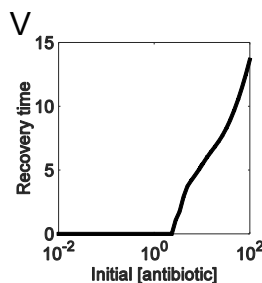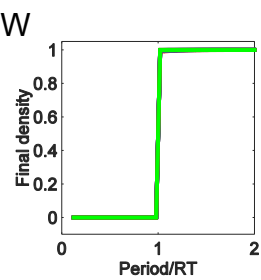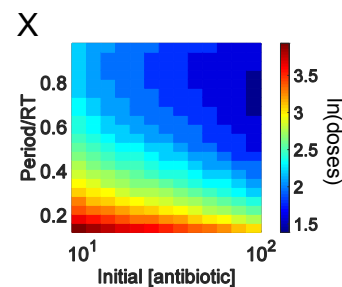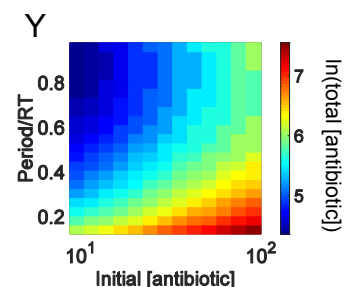

Supplement: S3 Fig — (A-E) No Bla production. (A) Time course. The recovery of bacteria that do not produce Bla depends on the intrinsic removal of the antibiotic caused by natural degradation and turnover by the body’s fluid. (B) Recovery time curve. Because the bacteria still undergo the process of lysing before recovering, the dosing protocol based on recovery time is still applicable. Without the production of Bla to aid in the recovery of the population, the recovery time is longer and monotonically dependent on the antibiotic concentration. (C) Final density. Protocols using periods of less than one recovery time are effective at eliminating the population, regardless of antibiotic concentration. (D) Dose number. For each antibiotic concentration and period combination, the corresponding number of doses necessary to eliminate the population was calculated. The regimens using antibiotics associated with longer recovery times require the fewest doses; however, low concentrations of antibiotic (a 0 ≈ 1) can be effective if applied at period lengths of 0.10–0.50 period/RT. (E) Total antibiotic delivered. Despite many different regimens requiring the same number of doses to clear an infection, the regimens could be differentiated by the total amount of antibiotic delivered. When comparing regimens with the same number of doses, the amount of antibiotics delivered decreases as the dose concentration decreases. (F-J) Low inducible Bla production. (F) Time course. The population recovers faster than a population that does not produce Bla because it generates sufficient Bla to degrade the antibiotic; however, the recovery time is slower than a population that constitutively produces Bla because more bacteria lyse before sufficient Bla accumulates to effectively remove the antibiotic. (G) Recovery time. The recovery time increases as the initial antibiotic concentration increases. (H) Final density. Protocols using periods of less than one recovery time are effective at clearing an infectio [file pcbi.1004201.s004.pdf]

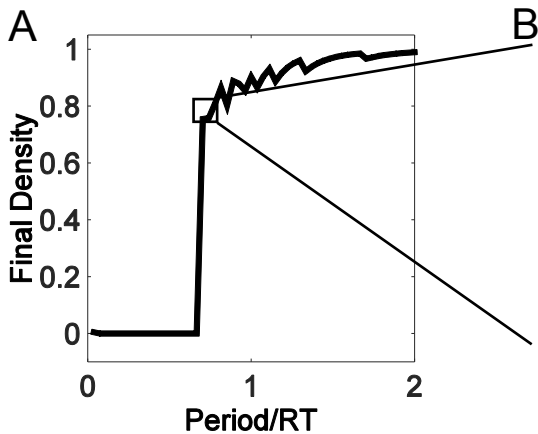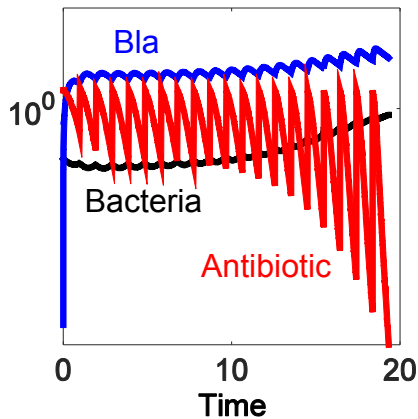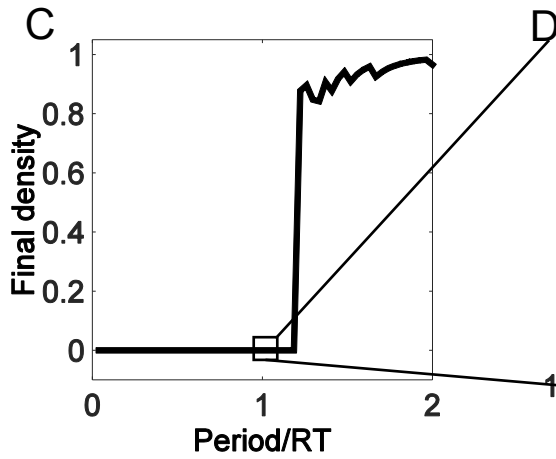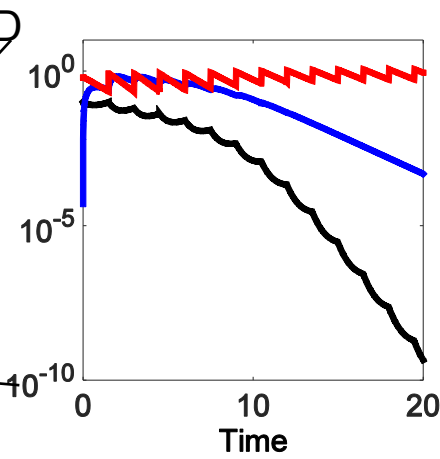

Supplement: S4 Fig — (A-B) Population recovers at periods < 1 recovery time. (A) Final Density. Populations can recover from an antibiotic applied at periods less than one recovery time if the bacteria are producing extreme levels of Bla and the antibiotic concentration is too weak to induce sufficient lysis (i.e. has a short recovery time). If the recovery time is too short, then the time between doses is too short for the Bla to return to a baseline level. As a result, the net Bla compounds with each subsequent dose, allowing populations to survive an antibiotic applied at periods less than one recovery time. (B). Time curves for cell density, Bla, and antibiotic concentration. Because each subsequent dose of antibiotic causes more cells to lyse and release Bla before the Bla from the previous dose is degraded, there is an increase in the base amount of Bla always present. As a result, the cells can clear the antibiotic faster on subsequent doses compared to the first dose. Consequentially, the observed recovery time from the multi-dose regimen is actually shorter than the expected response time calculated from the single dose. Here, A = 1.4 and period = 0.60 RT. (C-D) Population fails to recover at periods = 1 recovery time. (C) Final Density. Populations fail to recover from a low antibiotic concentration applied at periods less than one recovery time if the bacteria are producing low levels of Bla. Because the antibiotic concentration is low, the recovery time and its derived dosing periods are short. The time between doses is insufficient for the Bla to degrade enough of the antibiotic to recover. As a result, the antibiotic concentration compounds with each subsequent dose, preventing populations from recovering at periods greater than one recovery time. (D). Time curves for cell density, Bla, and antibiotic concentration. With each subsequent dose of antibiotic, more cells lyse and release Bla; however, the cells produce insufficient Bla to degrade the current dose of antibiotic [file pcbi.1004201.s005.pdf]

100x slower

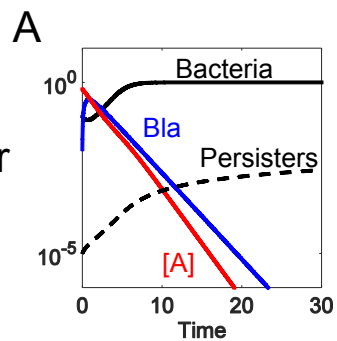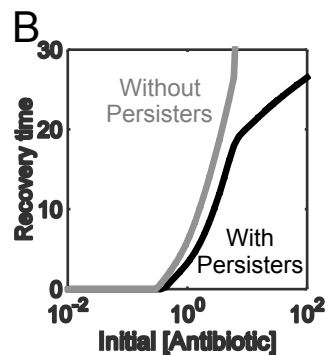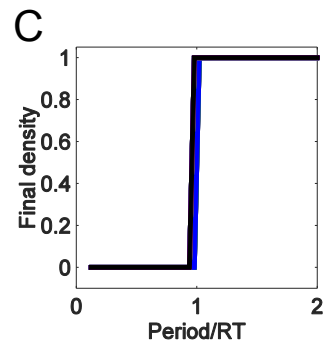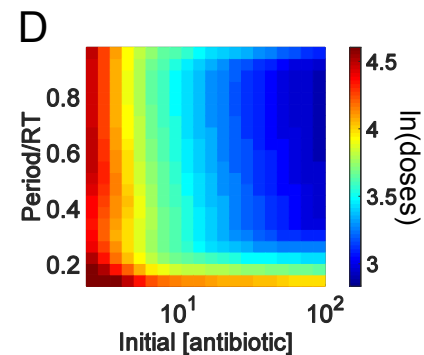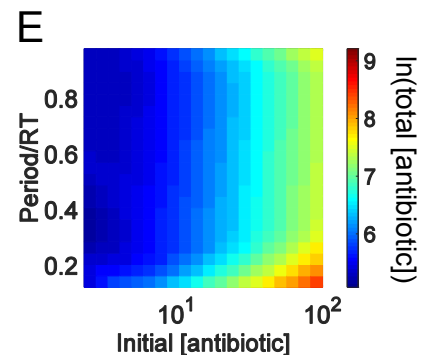

1000x slower

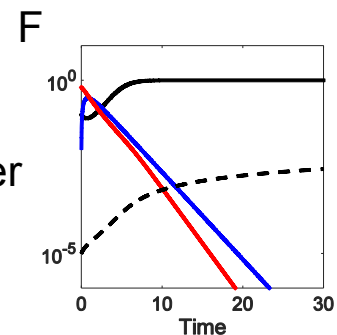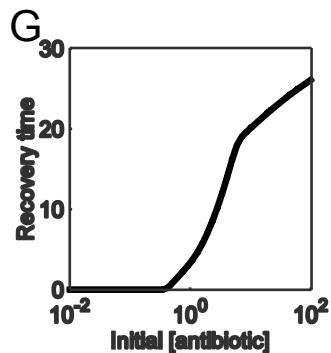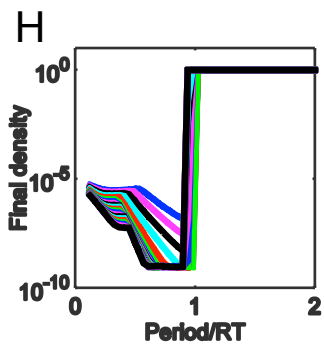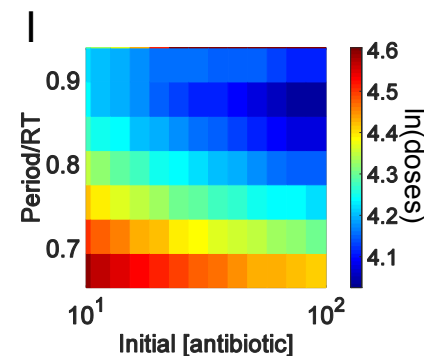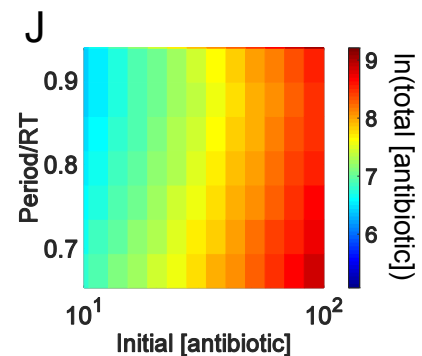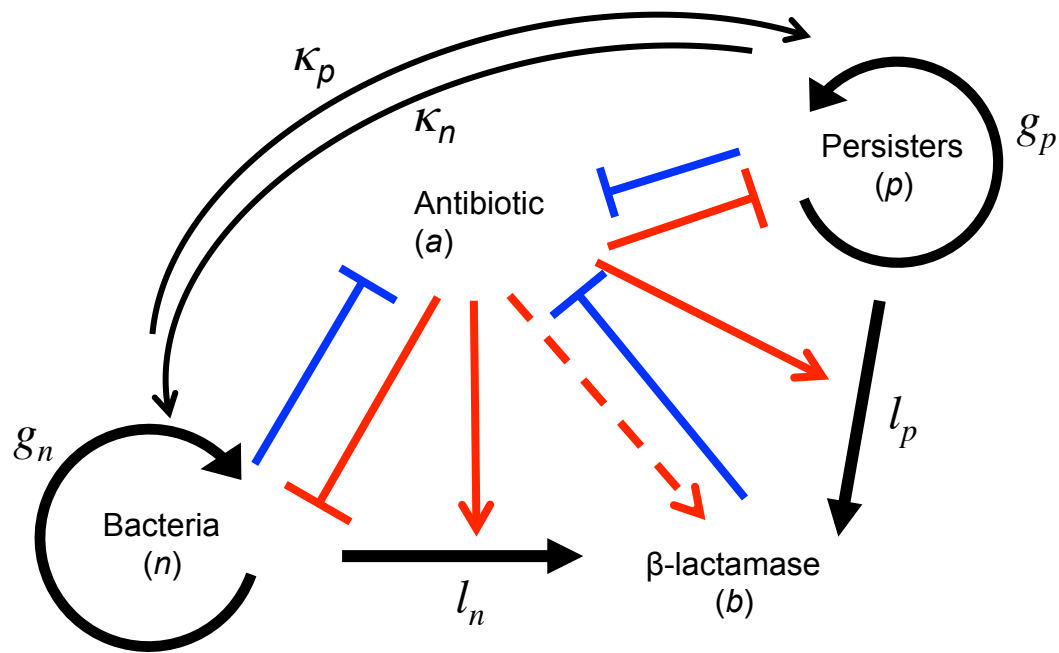

Supplement: S5 Fig — Our model assumes that persisters form a small fraction of a population, grow and lyse at rates much slower than normal cells (gp ≪ gN, lP ≪ lN), and, when the antibiotic concentration is low enough (a < σ1), they are generated from and revert to a normal cell phenotype at the slow rates of κP and κN, respectively. Population with persisters that grow and lyse at rates 100 times more slowly than normal cells (A-E). (A) Time course. The bacteria lyse due to the antibiotic, release Bla to degrade the antibiotic, and then recover once the antibiotic concentration is low enough. At this point, persisters (dashed black line) are generated from and return to the normal cell population (solid black line). Here, the initial density of persisters and normal cells are 0.00001 and 0.1, respectively. (B) Recovery time. The recovery times are the same between the population containing persisters (black line) and the population containing no persisters (grey line) until. a 0 > 0.3. From 0.3 < a 0 < 26, both populations take longer to recover with increasing antibiotic concentration; however, the population containing persisters recovers slightly faster. When a 0 > 26, then the population without persisters fails to recover, whereas the population with persisters is able to re-establish the population. (C) Final Density. After 100 doses of antibiotic, the final total density was measured. Periods greater than 1 recovery time resulted in the full recovery of the population; however, periods less than 1 recovery time appeared to suppress the population’s recovery. (D) Dose number. For each antibiotic concentration and period combination, the corresponding number of doses necessary to reduce the total population density to a critical threshold was calculated. The regimens using antibiotics associated with longer recovery times and longer periods require the fewest doses. (E) Total antibiotic delivered. Despite many different regimens requiring the same number of doses, the regimens c [file pcbi.1004201.s006.pdf]

Population switching

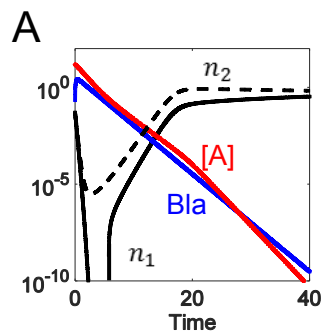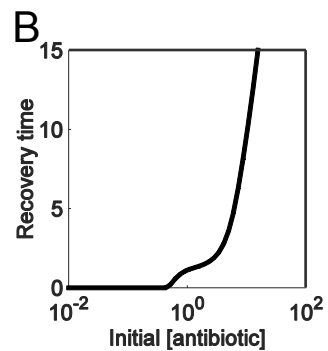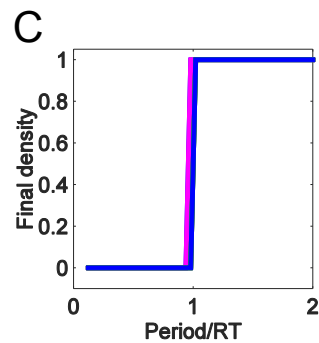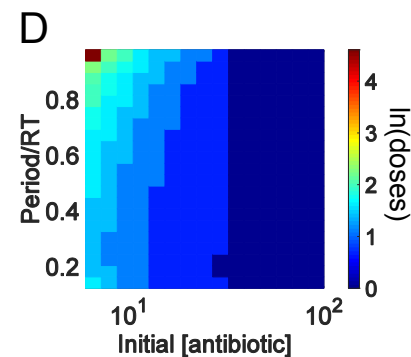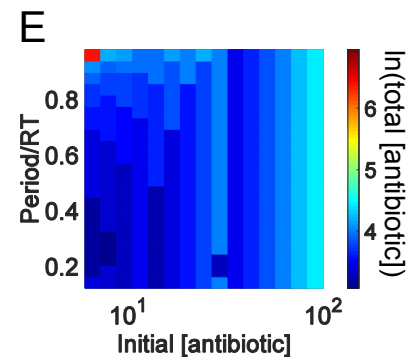

No population switching

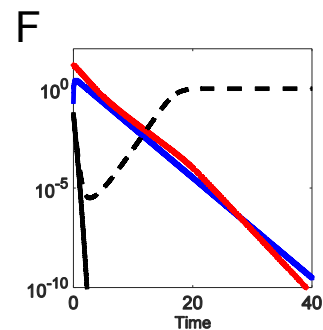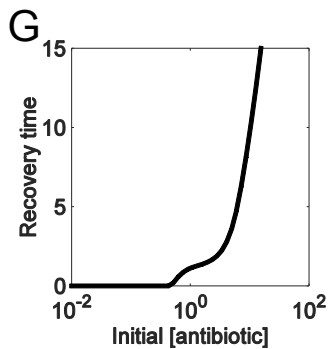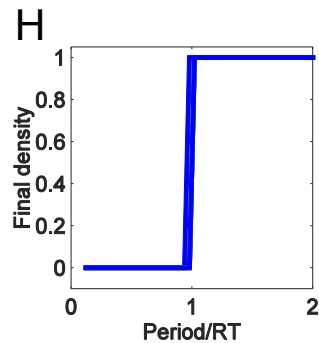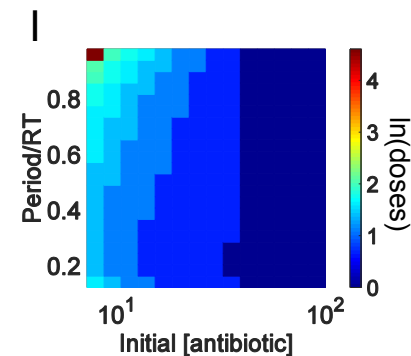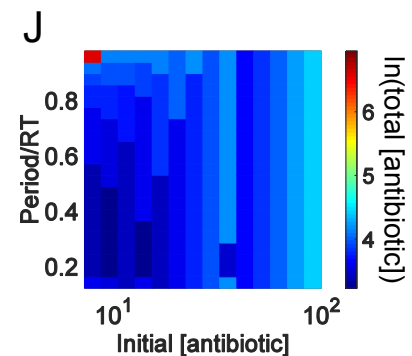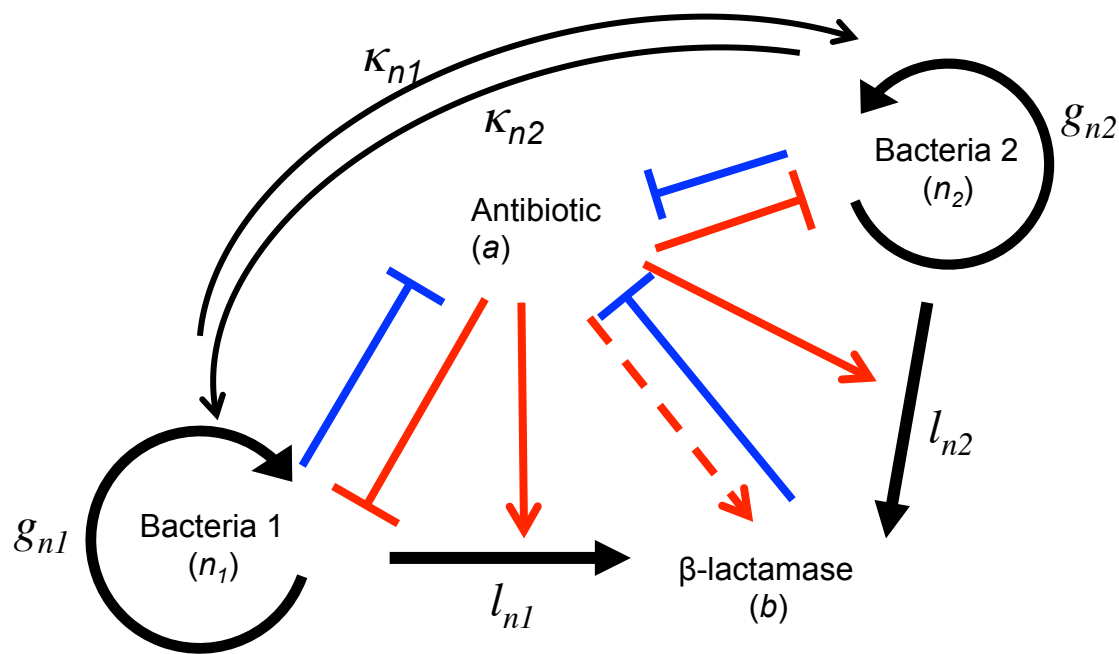

Supplement: S6 Fig — The model represents a mixed population with equal starting densities of two subpopulations (n 1 and n 2) with different levels of antibiotic resistance. n 1 has the same parameter values as the base case from the homogeneous model; however, n 2 has increased thresholds for antibiotic effects. Particularly, n 2 requires higher concentrations of antibiotic to inhibit growth (σ 5 = 5σ 1) and induce lysis (σ 6 = 5σ 2). Although this model accounts for two distinct subpopulations, it could be extended to multiple populations displaying some degree of collective antibiotic tolerance. Subpopulation can switch between states (A-E). (A) Time course. Both subpopulations start at the same starting density (0.05) and start to lyse due to antibiotic. One subpopulation (n 2) is more resistant than the other (n 1), with thresholds for growth inhibition (σ 5) and lysis (σ 6) 5 times higher. Both populations contribute Bla to degrade the antibiotic. Once the antibiotic concentration is sufficiently low, then the subpopulations can start to recover. Since n 1 and n 2 can switch between states, n 2 can help n 1 recover under concentrations that would otherwise be lethal. (B) Recovery time curve. Because both populations still undergo the process of lysing before recovering, the dosing protocol based on recovery time is still applicable. When 0.3 < a 0 < 2.3, both n 1 and n 2 are recovering at similar rates, thus the population as a whole recovers quickly. Once 2.3 < a 0 < 26, then n 2 recovers faster and determines the population level recovery time. When a 0 < 26, then both subpopulations cannot recover. (C) Final density. Protocols using periods of less than one recovery time are effective at eliminating the population, regardless of antibiotic concentration. (D) Dose number. For each antibiotic concentration and period combination, the corresponding number of doses necessary to eliminate the population was calculated. The regimens using antibiotics associated with longer recovery [file pcbi.1004201.s007.pdf]
